# Supplementary material for: Pervasive supply of therapeutic lysosomal enzymes in the CNS of normal and Krabbe‐affected non‐human primates by intracerebral lentiviral gene therapy
Source: EMBO Mol Med. 2016 Mar 29;8(5):489–510. doi: 10.15252/emmm.201505850 (PMC5128736; doi:10.15252/emmm.201505850)
Supplement: Supplementary file 1 — Appendix [file EMMM-8-489-s001.pdf]

Appendix

Table of contents

- **Appendix Supplementary Methods**
  - Mouse studies
  - In vitro studies
  - Surgical and post-surgical monitoring
  - Magnetic Resonance Imaging
  - Tissue collection and processing
  - Quantification of VCN
  - Western blot analyses
  - Histopathology
  - Primary and secondary antibodies
  - ELISA assay
  - p24 enzyme-linked immunosorbent assay

- **Appendix Supplementary Figures**
  - Appendix Fig. S1. Intracerebral injection of LV.hARSA in MLD mice.
  - Appendix Fig. S2. Post-surgery follow-up of LV.hARSA-injected NHP.
  - Appendix Fig. S3. ARSA expression in neurons and glial cells in the injected and contralateral hemisphere.
  - Appendix Fig. S4. VCN distribution in LV.hARSA-injected NHP.
  - Appendix Fig. S5. Expression of transgenic hARSA.
  - Appendix Fig. S6. Validation of LV.hGALC in vitro and in a Krabbe murine model.
  - Appendix Fig. S7. Necropsy and tissue collection of Krabbe-affected NHP

- **Appendix Supplementary tables**
  - Appendix Table S1. LV.GFP- and LV.ARSA-injected NHP
  - Appendix Table S2. Lentiviral vectors batches.
  - Appendix Table S3. CED parameters used for intracerebral infusion of LV suspension in normal NHP.
  - Appendix Table S4. Volume of injected vector suspension calculated based on post-surgery MRI scans.
  - Appendix Table S5. Score of pathological lesions in the injected areas of LV.hARSA-treated NHP.
  - Appendix Table S6. Gene Ontology (GO) analysis of genes targeted by LV integrations
  - Appendix Table S7. LV.hGALC-injected NHP and untreated controls
  - Appendix Table S8. Neurobehavioral assessment: modified infant neurobehavioral assessment scale (NBAS).
  - Appendix Table S9. Neurobehavioral assessment: modified Bayley's scale for infant development.
  - Appendix Table S10. Summary of statistics for the main figures and tables.

- **References**

Appendix Supplementary methods

Mouse studies

GALC-deficient Twitcher mice (mixed background of C57BL6 and FVB) and ARSA knockout mice (As2-/- or MLD mice) have been previously described (Lattanzi *et al*, 2010; Neri *et al*, 2011). Twitcher and MLD mice (males and females) were injected in the external capsule with LV.hGALC and LV.hARSA, respectively (single unilateral injection, $2x10^6$ TU/2μl) (Lattanzi *et al*, 2010). Mice were analyzed 20 days (Twitcher) or 3 months after the injection (MLD), evaluating efficiency of transduction (vector copy number by qPCR, immunoreactivity for the Myc peptide) and enzymatic activity (X-gal staining, GALC and ARSA assays) (Lattanzi *et al*, 2010) in CNS and non-CNS tissues. Age-matched untreated mutant and WT littermates were used as controls. All the animals used in the study were housed and bred in the animal facility of the San Raffaele Scientific Institute.

In vitro studies

Human fibroblasts derived from Krabbe disease-affected patients were obtained from the "Cell line and DNA Biobank from patients affected by Genetic Diseases", Istituto Gaslini, Genova, Italy. Neural stem cells from Twitcher mice were derived and cultured as previously described (Neri *et al*, 2011). Human Krabbe fibroblasts and Twitcher NSCs were transduced overnight with LV.hGALC at different MOI (from 10 to 100). Cells underwent three subculturing passages before being plated on matrigel-coated glass coverslips (30.000 cells/cm^2) and assessed for GALC activity by X-Gal staining (Neri *et al*, 2011). Untrasduced cells from sister cultures were used as negative controls.

Surgical and post-surgical monitoring.

LV.GFP and LV.hARSA-injected NHP. Surgical monitoring consisted of ECG, digital pulse oximetry, non-invasive blood pressure monitoring, heart rate and respiratory rate. Cage side observations were performed throughout the study to evaluate changes in general health, appearance, appetite and behavior: daily check of the animals was performed directly and using video monitoring. Any change in behavior was documented in the animal's clinical record. Neurobehavioral assessment was performed in LV.hARSA-injected animals prior to the implant surgery, 7-days after surgery and at 2-week intervals until the time of euthanasia. Features evaluated included the examination of the wound site, feeding, vomiting, menace, pupil size, gait and posture. Body weight assessment was performed on the day of implant surgery, and twice a week until the time of euthanasia.

LV.hGALC-injected NHP. Surgical monitoring consisted in ECG, digital pulse oximetry, non-invasive blood pressure monitoring, heart rate, end tidal CO2, and respiratory rate (Surgivet, Advisor Vital Signs Monitor). Body weight has been evaluated prior to infusion then weekly throughout study. Cage-side observations and neurological assessment have been performed prior to infusion and weekly after infusion until

necropsy. Neuromotor and behavioral assessments have been performed through the use of standardized behavioral profiles and clinical rating scales specifically designed for use with nonhuman primates. The behavioral staff at the TNPRC has used these profiles in rhesus monkeys for many years and is experienced in administering and interpreting them.

Modified Infant Neurobehavioral Assessment Scale (NBAS). This test is adapted for nonhuman primates from the Brazelton Neonatal Behavioral Assessment Scales used with humans (Brazelton, 1973). This test is valid for infants during the first four weeks of life. The scale consists of a 20-minute battery of tests that assess an infant's motor functioning, temperament, and interactive skills (Champoux *et al*, 2002a; Champoux *et al*, 2002b; Champoux *et al*, 1997). The 42 test items include numerous measurements that correspond to the typical course and manifestation of Krabbe disease, including visual orientation, state control, motor maturity, activity, reflexes and responses, fine and gross motor skills and strength, and temperamental items such as vocalization, self-quieting abilities, fearfulness, and distress. Scores are lumped into four categories for analysis: orientation, control, motor maturity, and activity. Prior research has found no sex differences in these measures so data from both males and females were included. Infants were evaluated using this scale at approximately 7 days, 14 days, and 28 days after birth. Composite scores, as well as individual items, were recorded for the 2 study animals (JT02 = Krabbe affected; JV02 = normal) as well as historical data collected on genetically normal animals and untreated, Krabbe-affected infants.

Modified Bayley scales of infant development. This test, originally developed for use with human infants (Bayley, 2000), has been modified for nonhuman primates between the ages of two months to one year (Champoux *et al*, 1994). This 10-minute test consists of 15 separate variables that are collapsed to three scores for analysis (problem-solving, motor abilities, and temperament). Beginning at two months of age, infants were evaluated monthly using this scale. For the study animals, scores were reported as pre-surgery and post-surgery. Historical data from animals of similar genetic status and age were collected and presented as means plus one standard deviation.

Magnetic Resonance Imaging

Magnetic Resonance Imaging (MRI) was performed pre-operatively (to determine surgical trajectories) and post-surgery (to assess specificity of injection, distribution of viral suspension and potential adverse events, i.e. hemorragia, edema). Animals were sedated in the surgery room with ketamine, a venous line was established using a catheter positioned in the saphenous vein. Animals were anesthetized using liquid anaesthesia ketamine (5-10 mg/kg) + propofol (2-6 mg/kg), placed in a stereotactic MRI compatible frame, transported to the MRI room and positioned in the scanner for the pre-operative MRI. The respiratory rate was continuously monitored during scanning (SA Instruments Inc., Stony Brook, NY, USA) and body temperature were maintained around 37°C using heated airflow. Total scan time for the pre-operative MRI was approximately 30 minutes. Upon

completion of the scan animals were immediately transferred to the operating room for the cranial implant procedure. After LV injection, animals were transferred back in imaging zone for post-injection MRI.
MRI acquisitions were performed on a whole-body horizontal 7T Agilent scanner (Palo Alto, CA, USA), using a surface coil for transmission and reception (RAPID Biomedical GmbH, Rimpar, Germany). T2-weighted images were acquired using a high-resolution 2D fast spin-echo sequence ($225 \times 225 \mu m^2$ in-plane resolution, and 1 mm slice thickness, 40 slices), with echo time TE/ repetition time TR=20/4750 ms, 5 echoes, effective TE=40 ms and acquisition time Tacq=16 min. Image analysis was performed using home made software BrainVISA (CEA, Orsay, France). LV volumes were measured using T2-weighted images. Both brain and injected volumes were manually segmented and reconstructed for 3D visualization.

Tissue collection and processing

Cerebral spinal fluid and blood samples were collected from each animal prior to surgery and at the time of euthanasia under ketamine/xylazine anesthesia.
At the day of euthanasia animals underwent deep anesthesia using Pentobarbital and cardiac perfusion with ice-cold PBS (600 ml per animal). Whole brain, cervical spinal cord, sciatic nerves, spleen, liver, gonads were collected.
For LV.GFP and LV.hARSA-injected NHP the brain was cut in 6 mm-thick slices using an adjustable brain matrix, obtaining 9-10 slices/brain (approximately 6 mm thick). Each slice was divided along the midline. Brain slices 2-7 were cut in 12 approximately equal specimens per hemisphere (16 blocks for NHP P1) and remaining slices were cut in squares of approx. 10x10 mm. A picture of each slice with its subdivision pattern was taken. Collected specimens were named using following scheme: number of slice (1 to 9-10), followed by right or left hemisphere (R or L) and by specimen number. Squares with numbers 3 (entire), 4 (half), 8 (half) and 10 (half) or 12 (half) of each hemisphere as well as samples of cervical spinal cord, left and right sciatic nerve, liver, spleen and gonads were fixed in PFA 4% and processed for histological analysis. Remaining brain samples were individually frozen in suitable cryoboxes on dry ice. An average of 102 blocks/hemisphere were retrieved (range 95-110) with an average volume of 0.32 cm^3/block.
For JV02 and JT02, the brain and cervical spinal cord have been removed and sectioned coronally into 3 mm-thick coronal sections. Slices have been divided along the midline in order to separate the injected from the non-injected hemispheres and blocks were cut following a 2 x 3 grid whenever possible. Blocks from **odd** slices have been immediately frozen in dry ice and stored for biochemical and molecular analysis. Blocks from **even** slices have been immersed in fixative (4% PFA) and processed routinely, embedded in paraffin or freezing compound (for storage at -80°C) to be used for histology (H&E), immunohistochemistry or immunofluorescence.
For all animals, cervical spinal cord was cut in 3-4 blocks that were frozen or fixed as described above.

Quantification of VCN

VCN in murine samples was quantified as previously described (Lattanzi *et al*, 2010). To quantify the VCN in NHP samples, standard curves were performed by using sequential dilutions of genomic DNA (100 ng to 0.8 ng) extracted from a human CEM cell clone carrying 1 LV integrated copy (CEM clone #25), previously validated by Southern blot analysis. To validate the accuracy of LV/TAF7-based system, sample of genomic DNA extracted from a human CEM cell clone carrying 6 LV integrated copies (CEM clone #37), previously validated by Southern blot analysis, were included in each run. Indeed, Taqman analysis, based on LV and TAF7 primers and probes and CEM25-based standard curve, detected ~5.8 VCN in CEM37 clone. Reactions were carried out in a total volume of 12.5μl, in a ViiA™ 7 Real-Time PCR System (Life Technologies-Applied Biosystems, Carlsbad, CA, USA).

The following criteria were applied to validate TaqMan experiments: i) standard curve: slope between 3.4 and 3.6, $r^2 > 0.95$; ii) CT of UT NHP sample > 37; iii) CEM37 VCN = 5.5±1.06. Only NHP samples with TAF7 probe CT within 22-26 were considered in the analyses. NHP samples with LV probe CT > 37 were considered with an undetectable VCN. Data were obtained by n=2 experiments performed in duplicate. Samples with intra-experiment CT variability > CT=2 (for TAF7 or LV) are excluded from the analyses. Samples with inter-experiment VCN variability > 50% are analyzed at least in n=3 experiments.

The VCN was calculated as follows: (ng LV/ng TAF7) × (number of LV integrations in the standard curve).

Western blot analyses

Tissues were homogenized with T10 basic ULTRA-TURRAX®, IKA in 500 μl of lysis buffer [PBS 1X, 50 mM TRIS HCl PH 7.4-7.5, 150 mM NaCl, 0,5% DOC, 0,1% SDS, 2mM EDTA, 1% Triton, protease inhibitor 7X (EDTA-Free Protease Inhibitor Cocktail, Roche) and phosphatase inhibitor 10X (PhosphoSTOP, Roche)], subjected to 3 rounds of sonication (three cycles of 15 pulses, Amplitude 0.7, 0.5 seconds oscillation) and to 3 freeze/thaw cycles (3' each). Lysates were centrifuged at 12.000 x *g* for 15' at 4°C, and supernatants were used as protein extracts for western blot analysis. We measured protein content using the Bradford Protein Assay kit with bovine serum albumin (BSA) as the reference standard. After boiling for 5 min in loading buffer (30% glycerol, 5% SDS, 9.25% Dithiothreitol, 1 μl of Bromophenol Blue, Tris-HCl 0.5 M, pH 6.8) samples containing 20-80 μg protein were separated through a 12% acrylamide gel SDS–PAGE electrophoresis. The transfer was performed at 400 mA for 1 hour and 30 minutes at 4°C. The PDVF membranes were then incubated in blocking solution of TBS-Tween 0.1% (Tris-Buffered Saline + Tween 20; TBS-T; Sigma Aldrich)+ 5% milk for 1 hour, and stained o/n at 4°C with primary antibodies diluted in TBS-Tween+3% milk solution. After 3 washes (10 minutes each), antibody staining was revealed using HRP-conjugated goat anti-rabbit (1:10.000; Chemicon) for 1 hour at RT in TBS-T+3% milk solution. Blots were developed with ECL system (Immobilon Western, Millipore) and were exposed to x-ray films (different exposure times according to the intensity of signals). Membranes were stripped for 15' with Stripping Buffer (Thermo Scientific), blocked and incubated with goat anti-β-actin antibody (1:10.000; Santa Cruz Biotechnology) and revealed with HRP-conjugated

chicken anti-goat (1:20.000; Santa Cruz Biotechnology). Primary antibodies: rabbit anti-GFP antibody (1:2.000; Molecular Probes); mouse anti-GFAP (1:100.000; Millipore), rabbit anti-Iba1 (1:2.000; Wako). The 27 KDa bands revealed using the anti-GFP antibody were quantified by means of ImageJ software and the values (in pixels) obtained were normalized on those of the corresponding b-actin band.

Histopathology

Post-fixed samples were processed into paraffin blocks. All the blocks from brain, spleen, gonads, liver, sciatic nerve and spinal cord were sectioned and stained in HE. This severity score in was attributed according to the size and number of observed lesions. Grade 0: within physiological limits. Grade 1: Minimal pathology generally affecting less than 1% of the tissue examined from a specific anatomic location. Grade 2: Mild pathology generally affecting 1-10% of the tissue examined from a specific anatomic location. Grade 3: Moderate pathology generally affecting 11-50% of the tissue examined from a specific anatomic location. Grade 4: Severe pathology generally with an overwhelming effect that involved more than 50% of the morphologic structure.

Primary and secondary antibodies

Immunofluorescence: mouse anti-GFAP (1:200; MAB3402, Chemicon-Millipore or C9205, Sigma Aldrich), rabbit anti-GFAP (1:100; ZO334, Dako), mouse anti-NeuN (1:100; MAB377, Chemicon), rabbit anti-Iba1 (1:200; 019-19741 Wako Chemicals), chicken anti-GFP (1:100; AB-13970b Abcam), mouse anti-CNPase (1:100; MAB326R Chemicon), mouse anti-APC (1:100; OP80 Calbiochem), mouse anti-ARSA (1:100; H00000410-B01P, Abnova); rabbit anti-ARSA (1:100; 19061-1-AP ProteinTech), mouse anti-GALC (1:100; H00002851-M01 Abnova), rabbit anti-GALC (1:100; H00002581-D01P, Abnova), rabbit anti-Myc tag (1:50 on tissues; 1:300 on cells; ab9106 Abcam), rabbit anti-S100β (1:1.000; S-2644 Sigma Aldrich). Secondary antibodies: Alexa 488-, Alexa 546-, Alexa 633- (Molecular Probes) conjugated anti-rabbit, anti-mouse, anti-rat, anti-chicken antibodies (1:1.000).

Images were taken using Leica Confocal SP2 microscope and Perkin Elmer UltraVIEW ERS Spinning Disk Confocal microscope. Images were imported into Adobe Photoshop CS4 (USA) and adjusted for brightness and contrast.

Immunohistochemisty: mouse monoclonal anti-CD3 (dilution: 1:400; Novocastra™), mouse monoclonal anti-CD20 (dilution: 1:800; Novocastra™) and mouse monoclonal anti-CD11c (1:100; Novocastra™), polyclonal anti-ARSA antibody (1:150; AF2485, R&D system)

ELISA assay

In each ELISA experiment (excluding ELISA assay to detect anti-hARSA antibodies) sera of 11 week-old BALB/c mice immunized by systemic injection of LV.ET.GFP.miR-142 (Annoni *et al*, 2009) were used as

positive controls (dilution of sera from 1:2*10^2 to 1:2*10^4). Sera of UT mice and pre-surgery NHP sera were used as negative controls. Standard curves based on serial dilutions (from 0.5 μg/ml to 0.03 μg/ml) of mouse IgG2a (BD Pharmigen) and Monkey IgG Isotype Control (Novus Biological) were used to calculate the antibody titers in mouse and NHP sera. The threshold was set as: (mean titer measured in pre-surgery NHP sera) + [(standard deviations) x 3]. Experiments (n=3) were performed in triplicates. Microplates (96-well) were coated with different antigens diluted in 1 M carbonate buffer (pH 9.5): i) recombinant-GFP, 0.3 μg/well (Vector Laboratories); ii) protein extracts of LV vector encoding for an unrelated protein (Factor IX), 2 μg/well; iii) protein extracts of 293T cells transfected with a plasmid over-expressing the p24 viral protein, 2 μg/well; iv) protein extracts of 293T cells transfected with a plasmid over-expressing the VSV-G viral protein, 2 μg/well. After three washes in PBS + 0.05% Tween-20, samples were incubated with blocking buffer (PBS + 0.05% Tween-20 + 1% ovalbumin) for 2 h at RT in the dark. Serial dilutions in PBS + 0.05% Tween-20 (from 1:2 to 1:2*10^4) of pre- and post-surgery sera of LV-injected NHP were added for 2 h at RT in the dark. Following four washes, anti-GFP, anti-LV, anti-p24 or anti-VSV-G were detected by adding 100 μl/well of diluted (1:2.000) peroxidase-conjugated goat anti-monkey IgA/IgG/IgM H/L chains (Novus Biologicals) for 1h at RT in the dark. After four washes, plates were reacted with OPD (Sigma–Aldrich) and H_2O_2 (final concentration 3%). The reaction was stopped by adding 50 μl/well of 1M sulfuric acid. Plates were read using an ELISA reader (VERSAmax Molecular Devices, Sunnyvale, CA, USA), and values are expressed as optical density (OD; λ 492 nm).

ARSA protein (concentration: 0.05, 1, 2 mU) purified by DEAE-cellulose chromatography was incubated in 96-well microplates (MaxiSorp™ , Nunc) in coating buffer (NaHCO3 0.1M pH 8.6) overnight at 4°C, after which LV.hARSA-injected NHP sera (dilution 1:2) were added and incubated overnight at 4°C. Following extensive washes with TBS+0.05% Tween-20, each well was exposed to anti-human peroxidase-conjugated secondary antibody (Sigma-Aldrich) and, subsequently, reacted with T0440 3,3′,5,5′-Tetramethylbenzidine (Sigma-Aldrich). Finally, absorbance was measured at λ 450 nm in Microplate Reader (GDV DV-990BV6). As quantification reference, a calibration curve was obtained by coating a serial dilution (from 0.016μg/ml to 2μg/ml) of the anti-human peroxidase-conjugated secondary antibody stained with T0440 3,3′,5,5′-Tetramethylbenzidine as described above.

p24 enzyme-linked immunosorbent assay

Upon protein lysis with 5% Triton X-100, serial dilutions of pre- and post-surgery sera were plated in triplicate in microplate wells coated with a mouse monoclonal antibody to the HIV-1 capsid protein p24. The captured antigen was then complexed with biotinylated polyclonal antibody to HIV-1 p24, followed by a streptavidin-HRP (horseradish peroxidase) conjugate. The resulting complex was detected by incubation with ortho-phenylenediamine-HCl, which produces a yellow color that is directly proportional to the amount of HIV-1 p24

captured. Absorbance was determined using a microplate reader (Versa Max, Molecular Devices) (λ 490 nm) and p24 amount was calculated by using an HIV-1 p24 antigen standard curve. Experiments (n=3) were performed in triplicates.

Appendix Supplementary figures

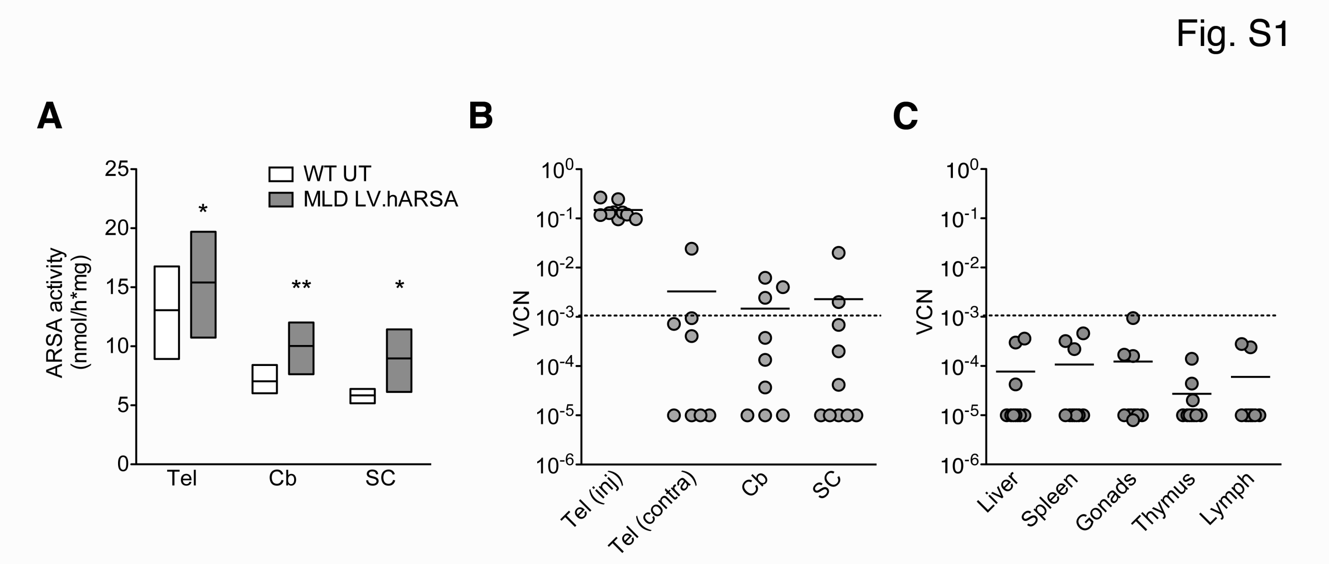

Appendix Figure S1. Intracerebral injection of LV.hARSA in MLD mice. LV.ARSA (2x10[6] TU/2 μl) was unilaterally injected in the external capsule (EC) of PND21 MLD mice. Mice were evaluated 3 months after injection for ARSA activity in CNS tissues and for integrated LV genome in CNS and non-CNS tissues. **(A)** LV.hARSA-injected MLD mice show supraphysiological levels of ARSA activity (assessed in age-matched WT littermates) in the telencephalon (Tel), cerebellum (Cb) and spinal cord. Age-matched untreated MLD mice show undetectable ARSA activity. Data are represented as floating bars (min to max, line at mean). n=3-6 animals/group. *p=0.04 (Tel), p=0.019 (SC), **p= 0.0043 versus WT UT, unpaired Student t-test. (**B**) Integrated LV genome is detected in the injected brain region (Tel inj) and, to a minor extent, in the contralateral hemisphere (Tel contra), Cb and SC. **(C)** VCN values below the threshold of detectability are found in liver, spleen, gonads, thymus and lymphnodes (lymph) of all injected mice. Each dot in (B) and (C) corresponds to one animal.

Fig. S2

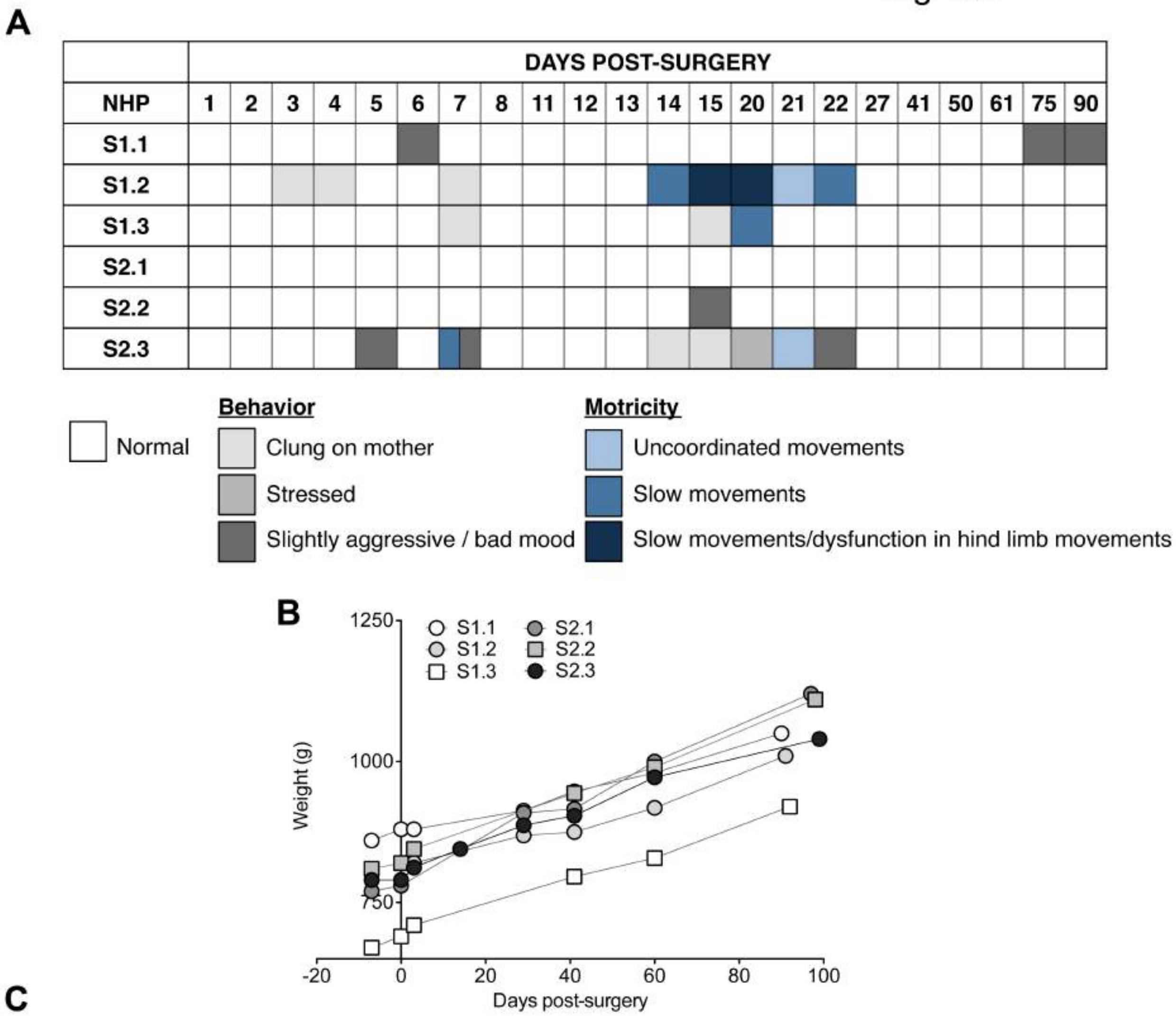

C

		Pre-surgery					Post-surgery										P value
		S1.1	*S2.2*	*U14*	***Mean***	***SEM***	*P1*	*P2*	*S1.1*	*S1.2*	*S2.1*	*S1.3*	*S2.2*	*S2.3*	***Mean***	***SEM***	P > 0.05
ALB	g/dL	4.1	3.7	4	***3.9***	***0.1***	3.3	4.6	3.6	4	3.4	4.4	3.6	3.7	***3.8***	***0.1***	P > 0.05
ALP	U/L	393	244	845	***494.0***	***180.7***	343	235	400	552	135	475	448	244	***354.0***	***49.8***	P > 0.05
ALT	U/L	36	36	21	***31.0***	***5.0***	18	34	23	17	16	19	16	36	***22.3***	***2.8***	P > 0.05
AST	U/L	57	59	52	***56.0***	***2.1***	62	70	112	40	36	79	65	59	***65.3***	***8.3***	P > 0.05
CK	U/L	1153	744	912	***936.3***	***118.7***	1048	2108	1207	573	431	968	754	744	***979.1***	***184.3***	P > 0.05
GLU	mg/dl	225	268	88	***193.6***	***54.3***	76	100	180	164	109	203	215	268	***164.3***	***23.1***	P > 0.05
LDH	U/L	1642	1164	1336	***1380.6***	***139.8***	1261	1256	1167	755	871	1048	830	1164	***1044.0***	***70.8***	P > 0.05
TRIG	mg/dL	75	53	63	***63.6***	***6.3***	45	55	77	32	39	48	38	53	***48.3***	***4.9***	P > 0.05

Appendix Figure S2. Post-surgery follow-up of LV.hARSA-injected NHP. (A) Daily check of the animals was performed directly and using video monitoring to evaluate changes in general health, appearance, appetite and behavior. Neurobehavioral assessments were performed prior to surgery (day 1) and continued until the end of experiment (day 90-96). Features evaluated included the examination of the wound site, feeding, vomiting,

menace, pupil size, gait and posture. **(B)** Body weight assessment was performed before surgery (day -7), at the day of surgery and twice a week until the end of experiment. **(C)** Serum chemistry was performed on samples collected pre-surgery and at the end of experiment (post-surgery). Pre-surgery serum collected from U14 NHP was included in the analysis. Data are expressed as mean ± SEM and analyzed by Two-way ANOVA and Bonferroni post-test (variables: analytes and treatment). $p>0.05$ for all analytes. ALB, albumin; ALP, alkaline phosphatase; ALT, alanine amino transferase; AST, aspartate amino transferase; CK, creatine kinase, GLU, glucose; LDH, lactic dehydrogenase; TRIG, triglycerides.

Fig. S3

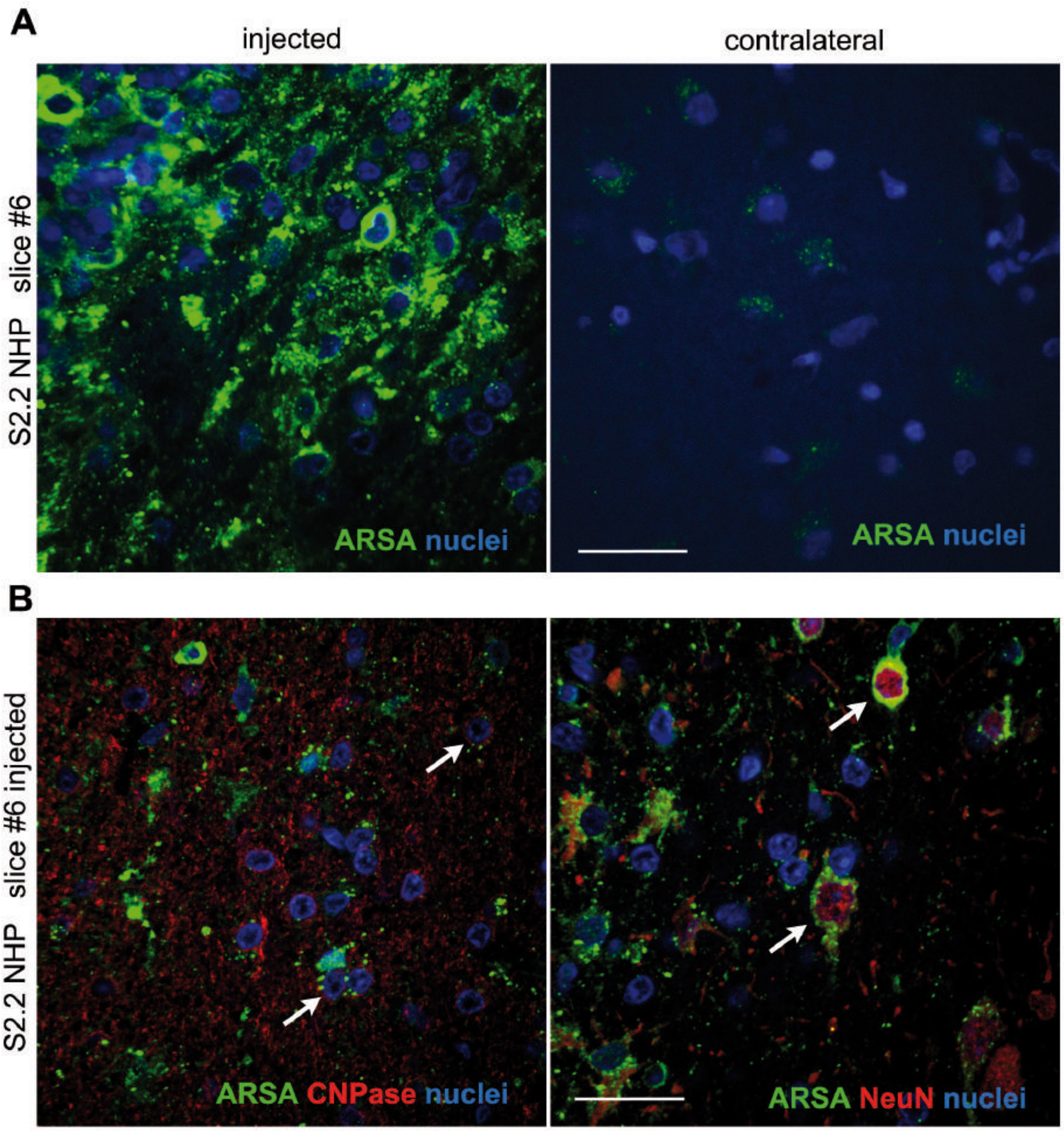

Appendix Figure S3. ARSA expression in neurons and glial cells in the injected and contralateral hemisphere. (A) Representative confocal images confocal images showing numerous ARSA-overexpressing cells close to the posterior injection site of S2.2 NHP (slice #6) and few cells showing weak ARSA immunoreactivity in the matched area of the contralateral hemisphere (putative cross-corrected cells). (**B**) Representative confocal images showing oligodendrocytes (CNPase, red) and neurons (NeuN, red) expressing

ARSA (green) close to the posterior injection site of S2.2 NHP. Arrows indicate co-localization of IF signals. Note the granular perinuclear ARSA staining, likely representing protein localized in the Golgi/lysosomal vesicles. In all pictures nuclei are counterstained with ToPro (blue). Scale bars: 60μm.

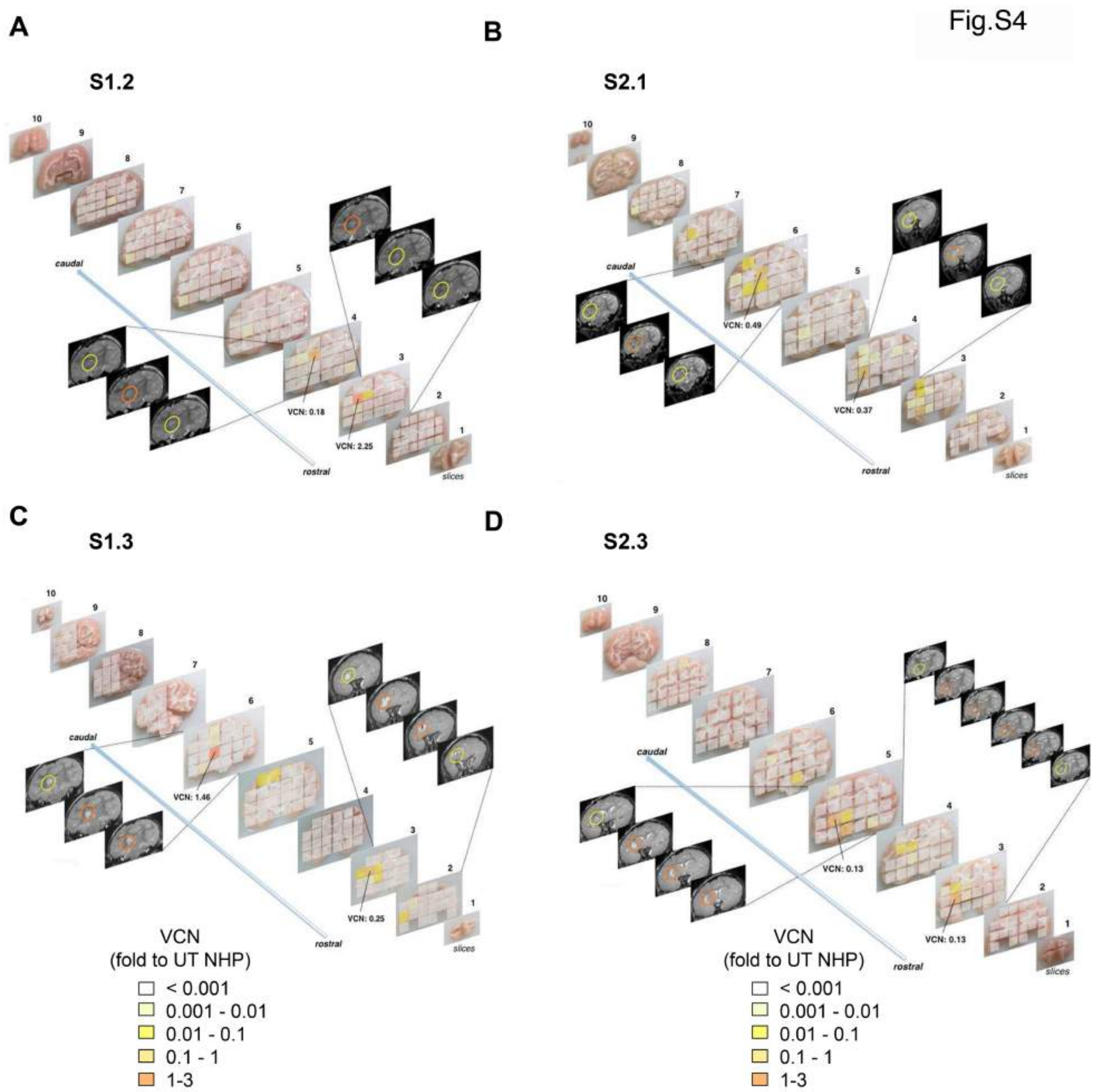

Appendix Figure S4. VCN distribution in LV.hARSA-injected NHP. (A-D) Vector copy number (VCN) cartography shows the distribution of integrated LV genome in the different blocks of brain slices 1 to 10 (rostral to caudal), assessed by qPCR analyses, of animals in study group 1 (S1.2, S1.3; **A, B**) and study group 2 (S2.1, S2.3; **C, D**). The color code indicates increasing VCN (lower threshold: VCN<0.001, corresponding to CT>37). The highest VCN is found in close correspondence to the injection sites, as confirmed by comparison with post-surgery MR images (yellow and orange circles indicate the presence of viral suspension around the injection sites, assessed 30-60 min after injection).

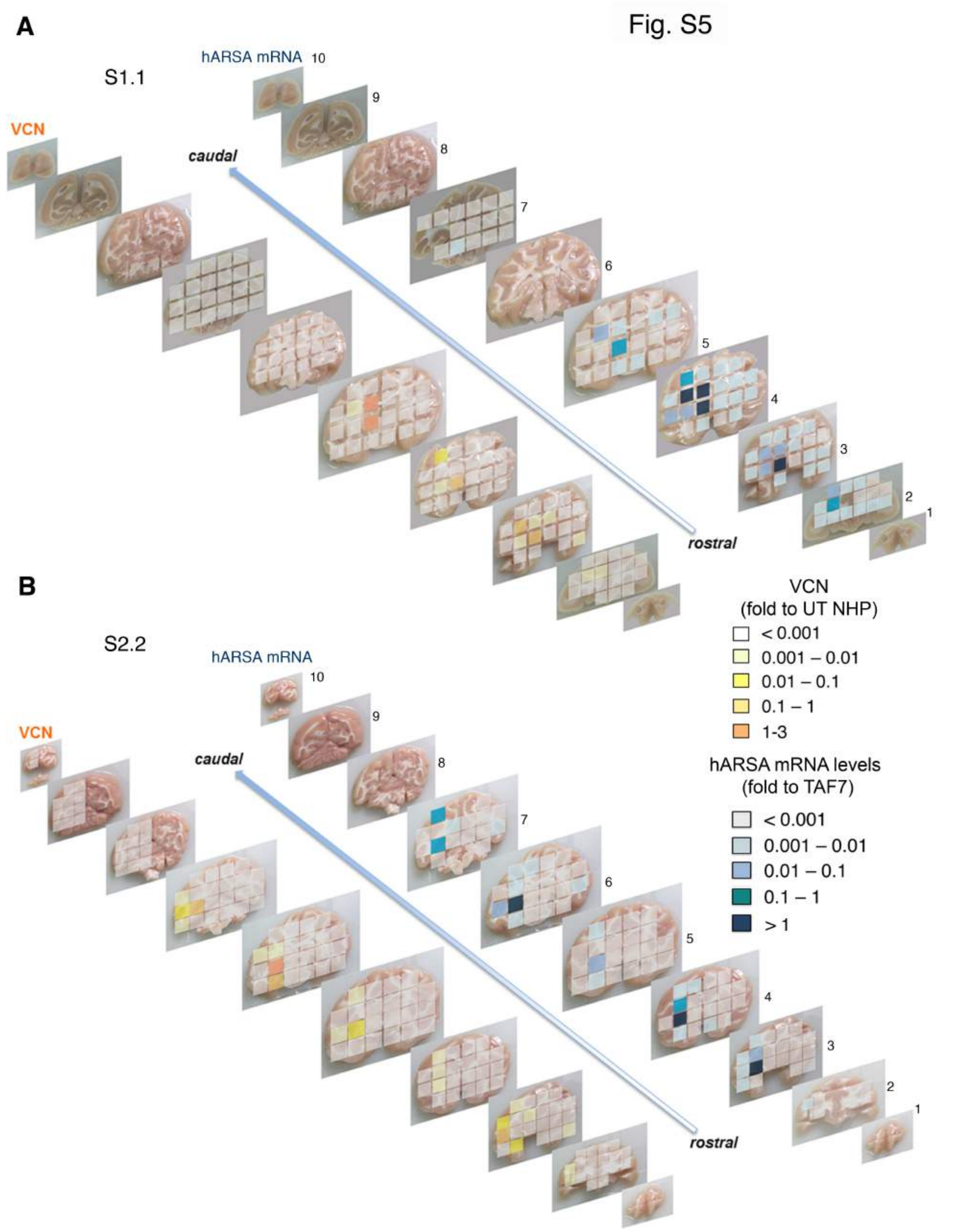

Appendix Figure S5. Expression of transgenic hARSA. Cartographies showing transgenic hARSA mRNA expression (quantified by using primers and probe annealing the wpre element in the 3'UTR of the LV.hARSA

cassette) in the brain of S1.1 NHP (**A**) and S2.2 NHP (**B**) in a side-by-side comparison with VCN distribution. ARSA mRNA levels are expressed as fold to TAF7 (normalizer). Grading scale of colors ranges from white (<0.001, corresponding to CT>37; undetectable expression) to dark blue (> 1 fold; robust expression). The volume of injected hemisphere with detectable transgenic hARSA mRNA was 28.6±0.9% and 30±7.3% (mean±SEM; n=3) of the total hemisphere volume in NHP of study group 1 and study group 2, respectively.

Fig. S6

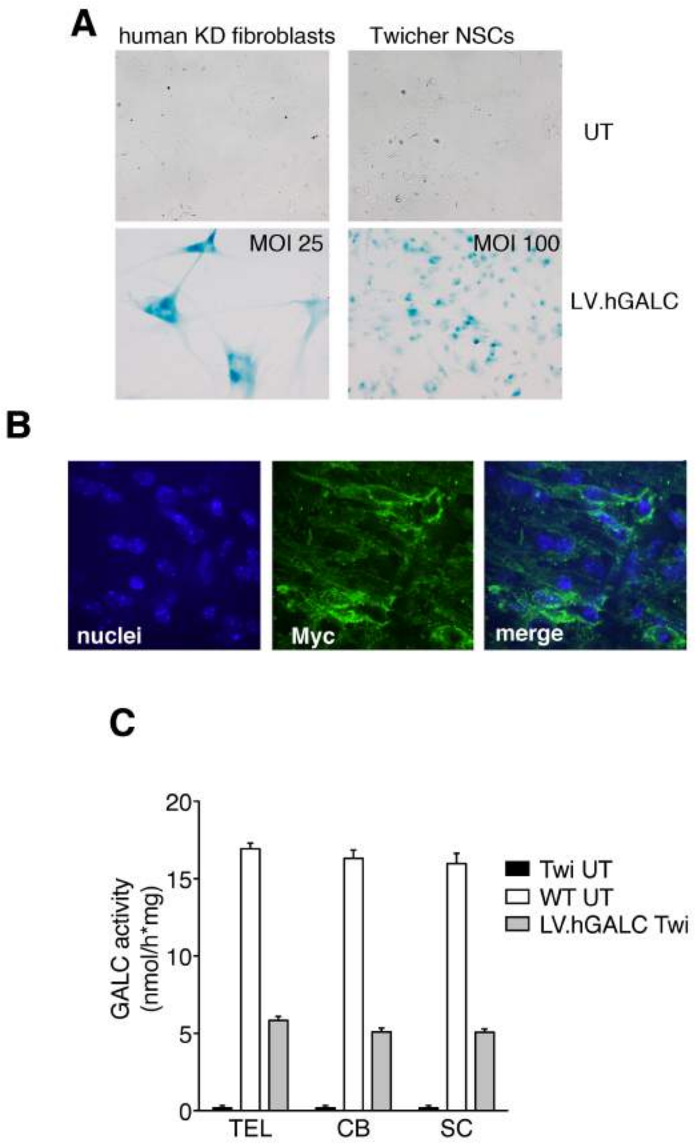

Appendix Figure S6. Validation of LV.hGALC in vitro and in a Krabbe murine model. (**A**) GALC activity assessed by colorimetric X-GAL staining in human fibroblasts from a Krabbe-affected patient and in neural stem cells (NSCs) from GALC-deficient Twitcher mice transduced with LV.hGALC at MOI 25 and MOI 100, respectively. UT, untreated**.** (**B**) Cells expressing GALC (identified by indirect IF using anti-Myc antibody) in the brain of Twitcher mice injected with LV.hGALC. Nuclei counterstained with ToPro. (**C**) GALC activity measured in the telencephalon (TEL), cerebellum (CB) and spinal cord tissues (SC) of Twitcher injected with LV.hGALC in the external capsule at PND2 and analyzed at PND40. Age-matched untreated (UT) WT and Twicher mice were uses as controls. Data are the mean± SEM; n=6-10 mice/group.

Fig. S7

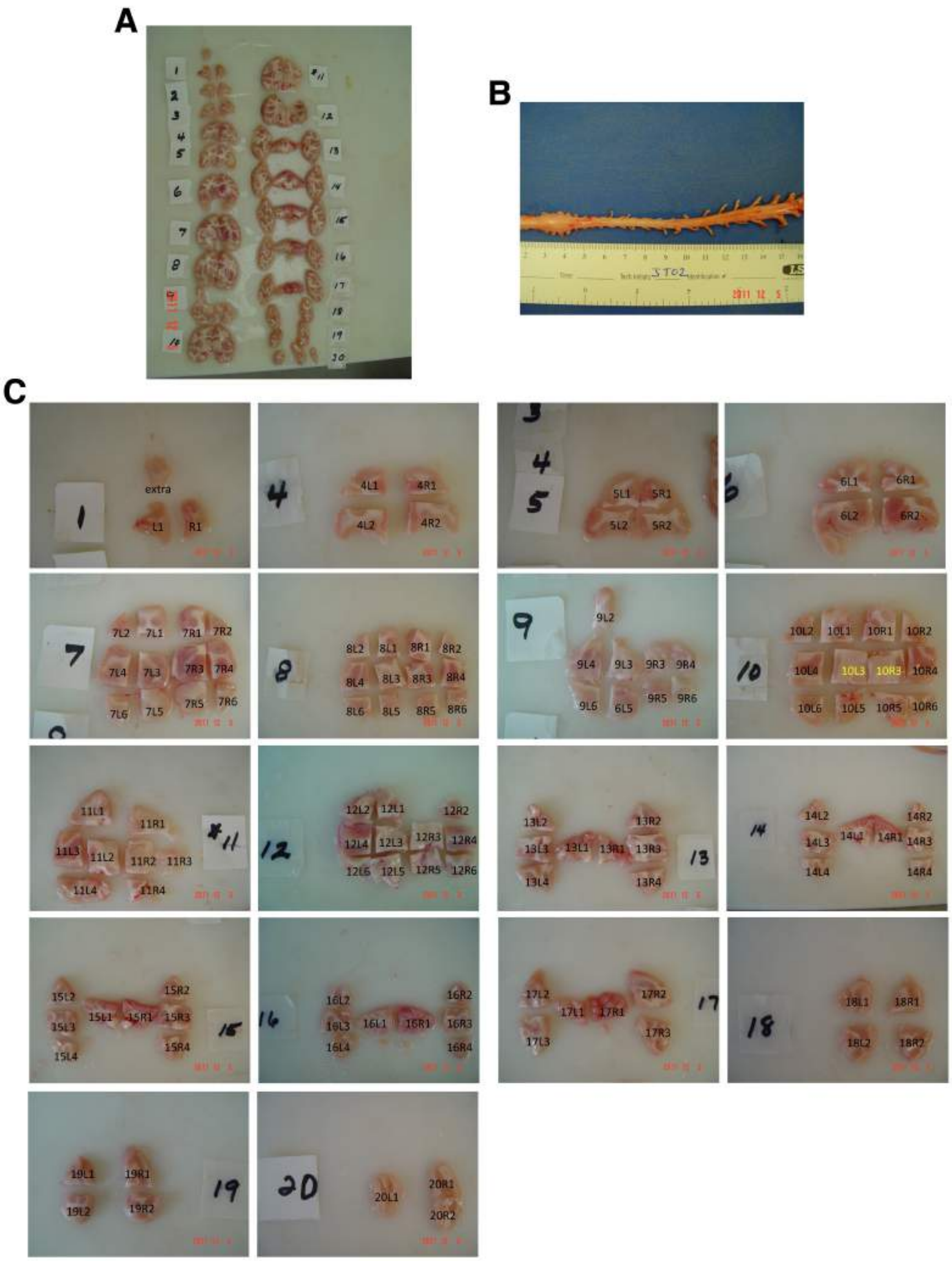

Appendix Figure S7. Necropsy and tissue collection of Krabbe affected NHP. At the end of experiment animals underwent deep anesthesia and were perfused with ice-cold PBS. **(A)** The whole brain was cut in 3 mm-thick slices, obtaining 19-21 slices/brain. **(B)** The cervical spinal cord was cut in 4 blocks. **(c)** Each brain slice was divided along the midline and each part subdivided in blocks. Yellow text in slice #10 indicates the block including the thalamic region in the injected hemisphere (right) and its matched block in the contralateral hemisphere (left).

Appendix Supplementary Tables

Appendix Table S1. LV.GFP and LV.ARSA-injected NHP. The table shows the ID (original and after recoding based on assignments to experimental groups), the gender, the age at surgery and at sacrifice of the nine juvenile *Macaca Fascicularis* used in the study. The asterisk indicates the animal that died from respiratory arrest (possibly due to an overdose of anesthetic) before surgery. This animal was not recoded and was perfused with saline, serum and brain tissues were collected, frozen and used as control untreated samples in several analysis.

Original ID	Recoded ID	Gender	Age at surgery (days)	Age at the end of experiment (days)
U7	P1	male	69	104
U8	P2	male	67	103
U13	S1.1	male	99	191
U14*	na	male	99	99
U15	S1.2	male	99	192
U16	S2.1	male	96	194
U17	S1.3	female	96	190
U18	S2.2	female	91	190
U19	S2.3	male	92	192

Appendix Table S2. Lentiviral vectors batches. The table shows titer, infectivity and endotoxin compliance of lentiviral vectors used in this study.

**Vectors**	**Titer (TU/ml)**	**Infectivity (TU/ng p24)**	**Endotoxin/TU ($\leq 1.25x10^{-7}$ EU/TU)**
LV.hARSA	$7.63x10^{8}$	$1.25x10^{5}$	$2.57x10^{-8}$
LV.GFP	$7x10^{9}$	$4.66x10^{4}$	nd
LV.hGALC-myc	$4.3x10^{9}$	$2.15x10^{4}$	nd

Appendix Table S3. CED parameters used for intracerebral infusion of LV suspension in normal NHP. The table reports the escalating infusion rate over time, the calculated and total infused volume (Vi) and the total time required for each injection site in animals of the pilot group and of the study groups.

PILOT GROUP

P1

Site 1 — Anterior External capsule

Infusion start time	rate (ul/min)	time (min.)	Vol.	Cum. Vol.
11:34	1	1	1	0
11:35	2	1	2	1
11:36	3	1	3	3
11:37	4	1	4	6
11:38	5	6	30	10
11:44	stop	na	na	40

Total time: 10 min
Vi (calculated): 40 ul
Total Vi (pump): 0.0406 ml

Site 2 — Thalamus

Infusion start time	rate (ul/min)	time (min.)	Vol.	Cum. Vol.
11:53	1	1	1	0
11:54	2	1	2	1
11:55	3	1	3	3
11:56	4	1	4	6
11:57	5	14	70	10
12:11	stop	na	na	80

Total time: 18 min
Vi (calculated): 80 ul
Total Vi (pump): 0.08 ml

P2

Site 1 — Anterior External Capsule

Infusion start time	rate (ul/min)	time (min.)	Vol.	Cum. Vol.
17:09	1	1	1	0
17:10	2	1	2	1
17:11	3	1	3	3
17:12	4	2	8	6
17:14	5	1	5	14
17:15	6	1	6	19
17:16	7	1	7	25
17:17	8	1	8	32
17:18	9	1	9	40
17:19	10	11	110	40
17:30	stop	na	na	150

Total time: 21 min
Vi (calculated): 150 ul
Total Vi (pump): 0.15 ml

Site 2 — Thalamus

Infusion start time	rate (ul/min)	time (min.)	Vol.	Cum. Vol.
16:05	1	1	1	0
16:06	2	1	2	1
16:07	3	1	3	3
16:08	4	1	4	6
16:09	5	28	140	10
16:37	stop	na	na	150

Total time: 32 min
Vi (calculated): 150 ul
Total Vi (pump): 0.15 ml

STUDY GROUP 1

Site 1 — Anterior External Capsule

Infusion start time	rate (ul/min)	time (min.)	Vol.	Cum. Vol.
11:10	1	1	1	0
11:11	2	1	2	1
11:12	3	1	3	3
11:13	4	1	4	6
11:14	5	14	70	10
11:28	stop	na	na	80

Total time: 18 min
Vi (calculated): 80 ul
Total Vi (pump): 0.08 ml

Site 2 — Thalamus

Infusion start time	rate (ul/min)	time (min.)	Vol.	Cum. Vol.
10:28	1	1	1	0
10:29	2	1	2	1
10:30	3	1	3	3
10:31	4	1	4	6
10:32	5	14	70	10
10:46	stop	na	na	80

Total time: 18 min
Vi (calculated): 80 ul
Total Vi (pump): 0.08 ml

STUDY GROUP 2

Site 1 — Anterior External Capsule

Infusion start time	rate (ul/min)	time (min.)	Vol.	Cum. Vol.
12:24	1	1	1	0
12:25	2	1	2	1
12:26	3	1	3	3
12:27	4	1	4	6
12:28	5	14	70	10
12:42	stop	na	na	80

Total time: 18 min
Vi (calculated): 80 ul
Total Vi (pump): 0.08 ml

Site 2 — Posterior External Capsule

Infusion start time	rate (ul/min)	time (min.)	Vol.	Cum. Vol.
11:52	1	1	1	0
11:53	2	1	2	1
11:54	3	1	3	3
11:55	4	1	4	6
11:56	5	14	70	10
12:10	stop	na	na	80

Total time: 18 min
Vi (calculated): 80 ul
Total Vi (pump): 0.08 ml

Appendix Table S4. Volume of injected vector suspension calculated based on post-surgery MRI scans. MRI was performed within 60 min from the end of surgery. Volumes (cm^3) were measured using T2-weighted images and expressed as percentages of mean hemisphere volume (32.5 cm^3).

ID	Experimental group	Vector	Volume of vector suspension in cm^3 (% of hemisphere volume)
**P1**	Pilot	LV.GFP	0.36 (1.1)
**P2**			0.36 (1.1)
**S1.1**	Study group 1	LV.hARSA	0.43 (1.3)
**S1.2**			0.43 (1.3)
**S1.3**			0.55 (1.7)
**S2.1**	Study group 2		0.70 (2.1)
**S2.2**			0.43 (1.3)
**S2.3**			0.43 (1.3)

Appendix Table S5. Score of pathological lesions in the injected areas of LV.hARSA-treated NHP. Histopathological lesions were observed in the injected hemisphere of five out of eight animals, including one animal with severe, two with moderate and two with mild inflammatory lesions, which included perivascular mononuclear cell infiltration, gliosis and mononuclear infiltration in the adjacent neuropil, intracytoplasmic eosinophilic accumulation. The score was attributed according to the size and number of observed lesions. Score: '0': within physiological limits, '1': minimal, '2': mild, '3': moderate, '4': severe. EC: External capsule; Thal: Thalamus; Ant: Anterior; Post: Posterior.

NHP	Perivascular infiltrations of mononuclear cells		Gliosis		Intracytoplasmic eosinophilic accumulations		Cumulative severity score
	Ant EC	*Thal*	*Ant EC*	*Thal*	*Ant EC*	*Thal*	
**P1**	2	2	2	2	2	2	mild
**P2**	2	2	2	2	2	2	mild
	Ant EC	*Thal*	*Ant EC*	*Thal*	*Ant EC*	*Thal*	
**S1.1**	0	0	0	0	0	0	physiological
**S1.2**	2	3	2	3	1	2	moderate
**S1.3**	0	0	0	1	0	0	physiological
	Ant EC	*Post EC*	*Ant EC*	*Post EC*	*Ant EC*	*Post EC*	
**S2.1**	0	0	0	0	0	0	physiological
**S2.2**	3	3	0	2	0	1	moderate
**S2.3**	4	4	4	3	3	1	severe

Appendix Table S6. Gene Ontology (GO) analysis of genes targeted by LV integrations following intracerebral LV injection. The datasets of genes targeted by LV integrations in brain tissues from normal NHP injected with LV.GFP (a) and LV.hARSA (b) were analyzed using online tool DAVID-EASE (http://david.abcc.ncifcrf.gov/) to score for the significant enrichment of specific gene classes adopting the default settings and filtering for significant results with a fold change ≥2. The first and second columns describe the gene ontology database used that provided significant enrichment results and the enriched gene class, respectively. Counts: number of genes targeted by LV integrations present within the specified gene class; Fold Change: change with respect the expected value. Benjamini: Adjusted *p* value (Benjamini-Hochberg; FDR: False Discovery Rate.

(a) LV.GFP-injected NHP (n=2)

GO term / Database	Gene class	Counts	Fold Change	Benjamini	FDR
**Cellular component**	**Synapse**	59	2.40	3.20E-07	8.50E-07
	**Synapse part**	46	2.70	3.90E-07	2.00E-06
	**Postsynaptic density**	20	4.10	3.80E-05	3.00E-04
	**Postsynaptic membrane**	28	3.00	6.10E-05	6.50E-04
**Molecular function**	**Glutamate receptor activity**	12	5.40	1.40E-03	8.80E-03
	**PDZ signaling domain**	28	2.70	8.50E-03	8.40E-03
	**GTPase regulator activity**	57	2.00	1.50E-03	2.30E-03
**INTERPRO**	**Laminin G, subdomain 2**	13	4.40	1.80E-02	3.70E-02
**SP_PIR_KEYWORDS (UNIPROT)**	**Synapse**	32	2.30	1.10E-03	3.70E-02
	**Postsynaptic cell membrane**	24	3.30	7.00E-05	8.80E-04
	**Cell junction**	52	2.00	2.80E-04	5.60E-03

(b) LV.hARSA injected NHP (n=2)

GO term / Database	Gene class	Counts	Fold Change	Benjamini	FDR
**Cellular component**	**neuron projection**	41	2.20	3.00E-04	5.40E-03
	**dendrite**	24	2.70	1.40E-03	3.40E-02
	**synapse**	53	2.70	2.30E-08	7.00E-08
	**synapse part**	41	3.10	9.10E-08	5.60E-07
	**postsynaptic membrane**	26	3.50	9.20E-06	8.40E-05
	**cell junction**	56	2.00	1.20E-04	1.90E-03
**Molecular**	**glutamate receptor activity**	12	6.90	4.00E-04	7.00E-04

**function**	**ionotropic glutamate receptor activity**	8	8.00	8.80E-03	4.70E-02
	**glutamate receptor activity**	12	6.90	4.00E-04	7.00E-04
**Biological process**	**synaptic transmission**	42	2.50	2.60E-04	1.60E-04
	**transmission of nerve impulse**	45	2.30	6.00E-04	7.60E-04
	**neuron development**	42	2.20	2.70E-03	5.10E-03
	**neuron projection morphogenesis**	29	2.40	1.10E-02	4.20E-02
**INTERPRO**	**Glutamate receptor, L-glutamate/glycine-binding**	8	8.00	1.40E-02	4.90E-02
	**Ionotropic glutamate receptor**	8	8.00	1.40E-02	4.90E-02
	**Glutamate receptor-related**	8	8.00	1.40E-02	4.90E-02
	**NMDA receptor**	8	8.00	1.40E-02	4.90E-02
	**Extracellular ligand-binding receptor**	11	5.50	2.50E-02	3.00E-02
	**Immunoglobulin I-set**	22	2.90	1.70E-02	4.00E-02
**SP_PIR_KEYWORDS (UNIPROT)**	**synapse**	33	2.90	1.40E-05	1.50E-04
	**postsynaptic cell membrane**	21	3.60	1.10E-04	1.80E-03
	**cell junction**	45	2.10	2.90E-04	5.50E-03
	**cell adhesion**	46	2.00	4.20E-04	1.00E-02
**UP_SEQ_FEATURE**	**binding site:Glutamate**	6	14.00	1.40E-02	3.90E-02
	**region of interest:Glutamate binding**	6	14.00	1.40E-02	3.90E-02

Appendix Table S7. LV.hGALC-injected NHP and untreated controls used in the study. The table shows the ID, genotype, gender, age at surgery and at sacrifice, and the treatment of the Krabbe affected rhesus monkeys used in the study.

ID	Genotype	Gender	Treatment	Age at surgery (days)	Age at the end of experiment (months)
JT02	Krabbe	male	LV.hGALC	53	4.6
JV02	WT	male	LV.hGALC	89	5.8
IG97	Krabbe	female	untreated	na	8
JN43	Krabbe	female	untreated	na	9
C180	Krabbe	female	untreated	na	6
KB76	Krabbe	female	untreated	na	8

Appendix Table S8. Neurobehavioral assessment: modified infant neurobehavioral assessment scale (NBAS). The table shows results on individual scale items as well as composite scores by testing age and group. The scale consists of a 20-minute battery of tests that assess an infant's motor functioning, temperament, and interactive skills. The 42 test items include numerous measurements that correspond to the typical course and manifestation of Krabbe disease, including visual orientation, state control, motor maturity, activity, reflexes and responses, fine and gross motor skills and strength, and temperamental items such as vocalization, self-quieting abilities, fearfulness, and distress. Scores are grouped into four categories: orientation control, motor maturity, and activity. Infants were evaluated using this scale at approximately 7 days, 14 days, and 28 days after birth. Scores are presented for the 2 study animals (JT02 = Krabbe affected; JV02 = normal) as well as historical data collected on genetically normal animals and untreated, Krabbe-affected infants (sample size for each group is indicated in the table). Historical data are presented as means plus one standard deviation.

	~7 days						~14 days						~28 days					
	Normal (n=2)		Affected (n=1)				Normal (n=16)		Affected (n=6)				Normal (n=22)		Affected (n=7)			
Individual Test Items	Mean	SD	Mean	SD	JT02	JV02	Mean	SD	Mean	SD	JT02	JV02	Mean	SD	Mean	SD	JT02	JV02
Initial State	2.00	0.00	2.00		2.00	3.00	1.75	0.43	2.00	0.00	2.00	2.00	2.09	0.61	2.14	0.83	2.00	2.00
Visual Orient	1.13	0.88	1.00		0.00	1.00	1.31	0.59	0.67	0.69	2.00	1.00	1.45	0.64	1.71	0.39	2.00	1.75
Visual Follow	2.00	1.00	2.00		0.50	1.00	1.50	0.94	0.92	1.17	3.00	1.00	2.07	0.99	2.86	0.23	3.00	2.50
Reach & Grasp	0.00	0.00	0.00		1.00	0.00	0.56	0.86	0.67	0.94	0.00	2.00	0.50	0.72	0.71	0.88	2.00	0.00
Startle to Auditory	0.00	0.00	0.00		1.00	0.00	0.69	0.92	0.42	0.73	3.00	0.00	0.64	0.71	0.71	1.03	0.00	0.00
Orient to Auditory	1.50	0.50	0.50		0.00	0.50	2.41	0.73	2.00	1.04	3.00	2.50	1.52	1.17	0.93	1.02	3.00	2.00
Duration of Looking	1.50	0.50	1.00		0.50	1.00	2.03	0.76	1.50	0.76	3.00	2.00	2.16	0.80	2.29	0.65	3.00	1.50
Distraction	1.00	1.00	0.50		2.00	0.00	0.97	0.57	1.58	0.61	1.00	1.00	1.09	0.60	0.86	0.83	0.00	1.00
Attention	1.50	0.50	2.00		1.00	2.00	1.72	0.56	1.08	0.73	2.00	2.00	1.70	0.54	1.71	0.45	2.00	2.00
Tacticle Response	0.25	0.00	0.50		0.75	2.00	1.02	0.65	0.75	0.38	0.75	2.00	0.86	0.46	1.18	1.01	1.25	1.00
Galants	0.25	0.25	2.00		1.00	2.00	1.03	0.89	0.08	0.19	1.00	2.00	1.09	0.78	0.93	1.02	0.50	1.00
Palmar & Plantar Grasp	2.38	0.63	2.00		2.50	3.00	2.52	0.50	2.63	0.54	2.50	3.00	2.55	0.40	1.89	0.55	2.00	2.00
Inversion	1.50	0.50	1.00		2.00	0.50	1.16	0.74	1.83	0.37	1.00	1.00	1.55	0.50	2.00	0.00	2.00	1.50
Head Posture Prone	1.50	0.50	1.00		1.00	1.00	1.63	0.48	1.33	0.47	1.00	1.00	1.89	0.37	1.00	0.76	2.00	2.00
Head Posture Supine	2.00	0.00	0.00		1.00	1.00	1.78	0.47	1.17	0.90	1.00	2.00	1.18	0.72	1.29	0.45	0.50	0.50
Body Righting	5.50	0.50	5.00		6.00	5.00	5.88	0.33	5.83	0.37	5.00	6.00	5.59	1.19	5.00	1.60	6.00	6.00
Body Righting - blindfold	5.50	0.50	2.00		6.00	6.00	5.00	1.70	5.33	1.49	6.00	5.00	5.00	1.53	4.57	1.68	5.00	6.00
Aversion to Back	1.50	0.50	1.00		2.00	1.00	1.44	0.61	1.58	0.45	2.00	2.00	1.45	0.66	1.57	0.49	2.00	2.00
Aversion to Blindfold	1.50	0.50	0.00		2.00	0.00	1.41	0.59	1.50	0.76	2.00	1.00	1.39	0.68	1.43	0.49	2.00	2.00
Traction	1.00	0.00	0.00		1.00	2.00	1.50	0.50	1.17	0.37	1.00	2.00	1.66	0.59	1.29	0.88	1.00	2.00
Labyrinthian Righting	1.50	0.50	2.00		0.00	0.00	1.41	1.16	0.25	0.56	2.00	1.50	1.80	1.15	0.57	0.68	1.00	1.00
Response Speed	1.50	0.50	1.00		1.50	1.50	1.66	0.70	1.67	0.69	2.00	2.00	2.16	0.46	1.64	0.35	2.00	2.00
Intensity (vocals)	1.00	0.00	0.00		0.00	1.00	0.78	0.83	1.08	0.61	1.00	0.50	0.93	0.59	1.43	0.68	0.50	2.00
Soothability	2.00	0.00	1.00		1.00	2.00	1.28	0.71	1.50	0.50	2.00	1.00	1.70	0.94	1.97	0.95	1.50	3.00
Cuddliness	2.00	0.00	2.00		2.00	2.00	1.81	0.39	2.00	0.00	2.00	2.00	1.59	0.72	1.67	0.47	2.00	1.00
Irritability/Distress	1.00	0.00	1.50		1.00	0.50	1.09	0.62	1.25	0.56	1.00	2.00	0.95	0.56	0.86	0.83	1.00	0.00
Consolability	2.00	0.00	2.00		1.00	1.50	1.75	0.43	1.67	0.47	1.00	2.00	1.48	0.61	1.36	0.58	2.00	0.00
Fearfulness	1.00	0.00	0.00		0.00	0.00	0.63	0.48	0.67	0.75	0.00	0.00	1.16	0.51	1.00	0.76	1.00	1.00
Self mouth	1.00	0.00	0.00		2.00	2.00	1.06	0.75	0.83	0.69	2.00	0.00	1.00	0.67	0.71	0.88	2.00	2.00
Struggle	1.00	0.00	0.00		1.00	1.00	0.75	0.66	0.83	0.69	1.00	1.00	1.39	0.93	0.79	0.75	0.50	2.00
Predominate State	2.50	0.50	1.00		2.00	2.00	2.00	0.59	1.67	0.47	2.00	2.00	2.45	0.52	2.64	0.95	2.00	3.50
Maintains Balance	2.00	0.00	0.00		1.00	1.50	2.13	0.60	1.83	0.37	2.00	2.00	2.59	0.49	2.00	0.93	2.00	2.50
Resistance - Passive MV	2.00	0.00	0.00		1.50	1.50	1.95	0.70	1.75	0.75	2.00	2.00	2.39	0.80	1.64	0.58	2.00	3.00
Active Power	2.00	0.00	0.00		1.50	1.00	1.75	0.53	1.42	0.45	1.50	2.00	1.98	0.49	1.36	0.44	1.50	2.00
Placing Response	1.00	0.00	0.50		0.50	1.00	0.97	0.08	1.00	0.00	1.00	1.00	0.98	0.10	0.86	0.23	1.00	1.00
Parachute	1.00	0.00	0.00		0.00	0.50	1.00	0.79	1.33	0.75	1.00	1.00	1.55	0.66	1.29	0.70	1.00	2.00
Rotation Test	1.75	0.25	1.50		1.50	1.50	2.00	0.00	1.42	0.53	2.00	2.00	1.66	41.00	1.79	0.36	2.00	1.50
Moro	1.00	0.25	0.00		0.50	0.50	0.58	0.26	0.75	0.20	0.75	1.25	0.70	0.42	0.57	0.46	0.50	1.00
Restrain	1.00	0.00	0.50		0.00	2.00	1.31	1.04	0.50	0.50	3.00	2.50	1.07	1.13	0.86	1.12	3.00	0.00
Persistence	1.00	0.00	0.50		0.00	1.50	1.00	0.79	0.50	0.50	2.00	2.00	1.05	1.07	0.71	0.88	3.00	1.00
Root	0.00	0.00	2.00		0.50	1.50	0.81	0.56	0.67	0.80	0.00	2.00	0.66	0.73	0.64	0.69	1.00	0.00
Composite Scores																		
Orientation	6.13	2.88	6.00		2.00	5.00	6.56	2.17	4.17	3.21	10.00	6.00	7.39	2.45	8.57	1.30	10.00	7.75
State Control	6.50	0.50	4.50		5.00	5.00	5.59	0.85	5.42	0.84	5.00	7.00	6.27	0.91	5.64	0.52	5.50	5.50
Motor Maturity	7.75	2.25	4.00		4.00	4.50	7.59	1.85	4.83	2.09	6.00	7.50	8.57	1.72	5.00	1.71	6.50	6.50
Activity	7.75	0.25	3.00		3.50	3.50	6.09	1.72	4.50	1.41	4.00	6.00	6.86	1.54	4.93	2.27	7.00	7.00
Grouped Items																		
Orientation	7.13	1.88	6.50		5.00	5.00	8.22	2.09	6.17	2.69	14.00	7.00	9.11	2.10	10.14	1.34	10.00	8.75
Neuromotor	12.25	1.75	5.00		6.00	6.50	11.34	2.57	7.83	2.59	9.00	10.50	13.07	1.93	8.43	2.86	10.50	11.00
Reflex	16.13	2.13	12.50		17.00	17.50	16.78	1.61	16.63	1.90	16.25	19.25	15.25	2.95	14.46	3.67	16.50	16.50
Muscle Tone	7.50	0.50	1.00		5.00	4.50	7.11	1.57	5.67	1.34	5.50	7.00	7.43	1.14	5.29	1.16	6.00	7.50
Temperament	6.00	0.00	3.00		2.00	5.50	5.09	2.14	5.00	1.12	7.00	6.00	5.82	2.13	6.11	1.75	7.00	6.00

Appendix Table S9. Neurobehavioral assessment: modified Bayley's scale for infant development. The table shows results on individual scale items as well as composite scores by testing age and group. The original scale developed for use with human infants has been modified for nonhuman primates between the ages of two months to one year. The 10-minute test consists of problem-solving, motor, and temperament tests. The 15 separate variables are collapsed to three scores for analysis (problem-solving, motor abilities, and temperament). Infants were evaluated monthly beginning at two months of age. Scores are presented for the 2 study animals (JT02 = Krabbe affected; JV02 = normal) as well as historical data collected on genetically normal animals and untreated, Krabbe-affected infants (sample size for each group is indicated in the table). For the study animals, scores are reported as pre-surgery and post-surgery. Historical data are presented as means plus one standard deviation.

	2 months							3 months						
	Normal (n=20)		Affected (n=12)		JT02			Normal (n=20)		Affected (n=10)			JV02	
Individual Test Items	Mean	SD	Mean	SD	Pre	Post	JV02	Mean	SD	Mean	SD	JT02	Pre	Post
Visual Orient	1.70	1.19	1.75	1.09	0.00	3.00	3.00	1.75	1.18	1.80	1.17	2.00	3.00	3.00
Lift Inverted Cup	0.85	0.73	0.63	0.84	1.00	0.00	0.00	0.55	1.02	0.50	0.81	0.00	0.00	0.00
Uncover Toy	0.45	0.50	0.50	0.65	2.00	0.00	0.00	0.75	1.09	0.50	0.67	2.00	0.00	3.00
Pull String to get toy	0.75	0.70	0.58	0.49	1.00	1.00	1.00	0.35	0.57	0.80	0.75	1.00	0.50	0.00
Remove Treat from bottle	0.98	1.01	0.33	0.75	1.00	2.00	0.00	0.55	0.92	0.30	0.64	0.00	0.00	0.00
Object Orientation	1.13	0.41	1.08	0.84	1.50	2.00	1.00	1.25	0.89	0.80	0.56	1.50	0.50	1.00
Distractability	1.20	0.60	0.96	0.88	0.00	0.00	1.00	0.95	0.82	1.35	0.78	0.00	2.00	2.00
Goal Directedness	0.93	0.51	0.63	0.51	1.00	1.00	0.50	0.60	0.66	0.55	0.47	0.50	0.00	1.00
Labyrinthian Righting	1.25	0.89	0.83	0.51	1.00	2.00	2.00	1.50	0.67	0.85	0.55	2.00	1.00	2.00
Muscle Tone	2.23	0.60	1.21	0.58	1.75	2.00	3.00	2.45	0.52	1.15	0.76	1.50	3.00	2.50
Active Power	1.33	0.55	0.54	0.43	1.50	1.00	1.50	1.55	0.52	0.65	0.50	0.75	2.00	1.50
Tension	2.10	0.44	1.17	0.82	2.00	1.00	3.00	2.30	0.56	1.53	0.90	1.00	3.00	3.00
Fine Motor Coordination	1.57	0.49	1.00	0.00	1.00	1.00		1.89	0.28	1.00	0.00	1.00	2.00	2.00
Responsiveness to Tester	1.73	0.99	1.75	1.09	0.00	0.00	3.00	1.60	0.97	1.70	0.90	0.00	3.00	3.00
Fearfulness	1.05	1.07	1.33	1.03	0.00	0.00	1.00	1.48	0.86	1.10	1.30	1.00	0.00	1.00
Irritability	1.55	1.07	1.92	1.38	1.00	1.00	3.00	1.23	1.05	2.70	0.64	0.00	3.00	3.00
Endurance	2.70	0.90	1.92	1.19	3.00	3.00	3.00	3.00	0.00	2.70	0.90	3.00	3.00	3.00
Activity	1.45	0.86	1.08	1.06	0.00	0.00	1.50	1.03	1.08	1.00	0.97	0.00	2.00	2.00
Response Intensity	0.95	0.72	1.50	0.45	0.00	1.00	1.50	0.60	0.60	1.45	0.65	0.00	1.50	2.00
Struggle	1.43	1.03	1.08	1.11	0.00	0.00	2.50	1.33	1.08	1.15	1.10	0.00	2.00	1.50
Consolability	1.35	0.55	1.09	0.70	0.00	0.00	2.00	1.38	0.57	1.15	0.81	0.00	2.00	2.00
Composite Scores														
Cognitive	6.78	2.41	5.50	3.52	7.50	9.00	5.50	5.80	3.72	5.25	3.25	7.00	4.00	8.00
Motor	6.90	1.72	3.75	2.01	6.25	6.00	9.50	7.80	1.33	4.18	2.43	5.25	9.00	9.00
Behavior	8.45	3.60	8.08	5.28	1.00	2.00	13.50	7.15	4.07	9.15	3.61	0.00	13.50	13.50

	4 months						5 months						6 months					
	Normal (n=13)		Affected (n=6)				Normal (n=6)		Affected (n=5)				Normal (n=1)		Affected (n=7)			
Individual Test Items	Mean	SD	Mean	SD	JT02	JV02	Mean	SD	Mean	SD	JT02	JV02	Mean	SD	Mean	SD	JT02	JV02
Visual Orient	2.31	0.99	1.83	1.21	3.00	0.00	2.83	0.37	1.20	1.17	0.00	1.00	2.00		1.57	1.05		3.00
Lift Inverted Cup	0.46	0.63	0.33	0.75	2.00	0.00	0.83	1.21	0.80	0.98	2.00	0.00	3.00		1.57	1.18		0.00
Uncover Toy	0.69	1.26	0.00	0.00	3.00	1.00	0.33	0.47	0.80	1.17	2.00	0.00	1.00		0.71	1.03		0.00
Pull String to get toy	0.23	0.42	0.75	0.80	1.00	0.00	1.00	1.00	0.60	0.49	2.00	0.00	1.50		0.71	0.70		0.00
Remove Treat from bottle	0.23	0.58	0.33	0.75	2.00	0.00	0.33	0.75	0.00	0.00	2.00	0.00	2.00		1.29	0.88		0.00
Object Orientation	1.04	0.69	0.67	0.75	3.00	0.00	1.08	0.98	0.90	0.80	2.50	0.00	2.50		1.57	0.68		0.00
Distractability	1.19	0.91	1.50	0.76	0.00	2.00	1.33	0.94	1.40	0.80	0.00	2.00	1.00		0.79	0.75		2.00
Goal Directedness	0.62	0.81	0.67	0.75	3.00	0.00	0.75	0.90	0.60	0.49	2.00	0.00	2.00		0.93	0.42		0.00
Labyrinthian Righting	1.62	0.62	0.67	0.94	2.00	1.00	1.33	0.75	0.60	0.80	2.00	0.00	2.00		1.00	0.76		0.50
Muscle Tone	2.42	0.47	1.21	0.58	1.50	3.00	2.75	0.56	1.20	0.66	2.00	3.00	3.00		1.00	0.57		3.00
Active Power	1.65	0.46	0.92	0.61	0.50	2.00	1.75	0.56	0.70	0.51	1.50	2.00	2.00		0.71	0.36		2.00
Tension	2.54	0.63	1.75	1.22	2.00	3.00	2.67	0.47	1.40	0.73	2.00	3.00	2.00		1.36	0.44		4.00
Fine Motor Coordination	1.94	0.17	1.00	0.82	1.00	2.00	2.00	0.00	0.75	0.43	1.00		2.00		0.90	0.20		2.00
Responsiveness to Tester	1.54	1.01	1.17	1.21	0.00	3.00	2.00	0.82	1.60	0.49	0.00	3.00	2.00		1.43	0.90		3.00
Fearfulness	2.23	1.31	0.67	1.11	1.00	0.00	1.50	1.38	0.40	0.49	1.00	1.00	0.00		0.86	0.64		1.00
Irritability	1.54	1.08	2.25	1.07	0.00	3.00	2.00	1.15	3.00	0.00	0.00	3.00	3.00		2.29	0.88		2.50
Endurance	3.00	0.00	2.50	1.12	3.00	3.00	3.00	0.00	2.20	1.17	3.00	0.00	3.00		2.14	1.36		3.00
Activity	1.35	0.95	0.92	1.02	0.00	3.00	1.83	1.07	0.80	0.75	0.00	3.00	1.50		0.71	0.70		3.00
Response Intensity	0.96	0.80	1.17	0.37	0.00	2.00	1.33	0.75	1.80	0.40	0.00	1.00	2.00		1.29	0.52		2.00
Struggle	1.42	0.96	1.17	1.34	0.00	3.00	1.75	1.15	0.60	0.80	0.00	3.00	0.00		0.57	0.49		3.00
Consolability	1.31	0.61	1.33	0.75	0.00	2.00	0.67	0.75	1.50	0.45	0.00	2.00	1.50		1.00	0.76		2.00
Composite Scores																		
Cognitive	5.58	3.76	4.58	3.03	17.00	1.00	7.17	4.31	4.90	3.04	12.50	1.00	14.00		8.36	3.99		3.00
Motor	8.23	1.12	4.54	2.35	6.00	9.00	8.50	2.06	3.90	1.83	7.50	8.00	9.00		4.07	1.08		9.50
Behavior	8.12	3.49	8.00	3.93	0.00	16.00	9.58	3.35	9.30	1.17	0.00	15.00	10.00		7.29	2.93		15.50

Appendix Table S10. Summary of statistics for the main figures and tables

Figure	Statistical analysis				
**Fig.4C**	**Two-way ANOVA**				
	Source of Variation	% of total variation	P value		
	Interaction	2.52	0.3201		
	region	83.43	< 0.0001		
	group	3.43	0.0371		
	Source of Variation	P value summary	Significant?		
	Interaction	ns	No		
	region	***	Yes		
	group	*	Yes		
	Source of Variation	Df	Sum-of-squares	Mean square	F
	Interaction	3	0.02752	0.009174	1.264
	region	3	0.9119	0.304	41.88
	group	1	0.03753	0.03753	5.171
	Residual	16	0.1161	0.007257	
	Bonferroni posttests				
	Physiological vs TOTAL BRAIN				
	group	Physiological	TOTAL BRAIN	Difference	95% CI of diff.
	GROUP 1	1	1.301	0.3014	0.09213 to 0.5106
	GROUP 2	1	1.194	0.1944	-0.01480 to 0.4037
	group	Difference	t	P value	Summary
	GROUP 1	0.3014	4.333	P<0.01	**
	GROUP 2	0.1944	2.795	P < 0.05	*
	Physiological vs INJECTED				
	group	Physiological	INJECTED	Difference	95% CI of diff.
	GROUP 1	1	1.505	0.5046	0.2953 to 0.7138
	GROUP 2	1	1.471	0.4706	0.2613 to 0.6798
	group	Difference	t	P value	Summary
	GROUP 1	0.5046	7.254	P<0.001	***
	GROUP 2	0.4706	6.766	P<0.001	***
	Physiological vs CONTRALATERAL				
	group	Physiological	CONTRALATER	Difference	95% CI of diff.
	GROUP 1	1	1.123	0.1228	-0.08644 to 0.3321
	GROUP 2	1	0.9474	-0.05263	-0.2619 to 0.1566
	group	Difference	t	P value	Summary
	GROUP 1	0.1228	1.766	P > 0.05	ns
	GROUP 2	-0.05263	0.7567	P > 0.05	ns

Figure	Statistical analysis				
Fig.5B	**Two-way ANOVA**				
	UT/LV.GFP vs S1.1 injected				
	Source of Variation	% of total variation	P value		
	Interaction	6.56	0.012		
	hem	43.61	< 0.0001		
	slice	12.17	< 0.0001		
	Source of Variation	P value summary	Significant?		
	Interaction	*	Yes		
	hem	***	Yes		
	slice	***	Yes		
	Source of Variation	Df	Sum-of-squares	Mean square	F
	Interaction	14	475.3	33.95	2.171
	hem	2	3159	1580	101
	slice	7	881.7	126	8.054
	Residual	131	2049	15.64	
	Bonferroni posttests				
	slice	UT/LV.GFP	S1.1 INJECTED	Difference	95% CI of diff.
	2	29.92	37.36	7.435	-2.707 to 17.58
	3	29.74	45.35	15.61	9.903 to 21.32
	4	29.28	40.17	10.89	3.637 to 18.14
	5	29.24	39.31	10.06	2.812 to 17.32
	6	30.38	40.15	9.776	3.516 to 16.04
	7	31.15	39.94	8.788	2.577 to 15.00
	8	31.71	45.63	13.92	5.134 to 22.70
	9	32.38	50.36	17.98	7.838 to 28.12
	slice	Difference	t	P value	Summary
	2	7.435	2.303	P > 0.05	ns
	3	15.61	8.591	P<0.001	***
	4	10.89	4.716	P<0.001	***
	5	10.06	4.358	P<0.001	***
	6	9.776	4.905	P<0.001	***
	7	8.788	4.444	P<0.001	***
	8	13.92	4.977	P<0.001	***
	9	17.98	5.568	P<0.001	***

**Two-way ANOVA**				
UT/LV.GFP vs S1.1 contra				
Source of Variation	% of total variation	P value		
Interaction	15.33	0.0014		
hem	13.39	< 0.0001		
slice	27.93	< 0.0001		
Source of Variation	P value summary	Significant?		
Interaction	**	Yes		
hem	***	Yes		
slice	***	Yes		
Source of Variation	Df	Sum-of-squares	Mean square	F
Interaction	7	268.8	38.4	3.76
hem	1	234.8	234.8	22.99
slice	7	489.6	69.95	6.849
Residual	79	806.8	10.21	
Bonferroni posttests				
slice	UT/LV.GFP	S1.1 CONTRAL	Difference	95% CI of diff.
2	29.92	29.91	-0.006664	-7.338 to 7.324
3	29.74	31.66	1.914	-2.448 to 6.277
4	29.28	31.43	2.152	-3.644 to 7.947
5	29.24	32.7	3.456	-2.042 to 8.954
6	30.38	32.25	1.878	-2.854 to 6.610
7	31.15	31.73	0.5775	-3.912 to 5.067
8	31.71	34.8	3.092	-3.257 to 9.440
9	32.38	47.52	15.14	7.812 to 22.47
slice	Difference	t	P value	Summary
2	-0.006664	0.002554	P > 0.05	ns
3	1.914	1.233	P > 0.05	ns
4	2.152	1.043	P > 0.05	ns
5	3.456	1.766	P > 0.05	ns
6	1.878	1.115	P > 0.05	ns
7	0.5775	0.3614	P > 0.05	ns
8	3.092	1.368	P > 0.05	ns
9	15.14	5.804	P<0.001	***

UT/LV.GFP vs S1.2 injected				
Source of Variation	% of total variation	P value		
Interaction	2.53	0.3435		
hem	60.02	< 0.0001		
slice	5.34	0.0813		
Source of Variation	P value summary	Significant?		
Interaction	ns	No		
hem	***	Yes		
slice	ns	No		
Source of Variation	Df	Sum-of-squares	Mean square	F
Interaction	3	23.23	7.744	1.145
hem	1	551.7	551.7	81.56
slice	3	49.06	16.35	2.417
Residual	38	257.1	6.765	
Bonferroni posttests				
slice	UT/LV.GFP	S1.2 injected	Difference	95% CI of diff.
3	29.74	38.47	8.727	5.290 to 12.16
4	29.28	36.74	7.46	2.885 to 12.04
5	29.24	34.16	4.918	0.3424 to 9.493
6	30.38	38.97	8.591	4.044 to 13.14
slice	Difference	t	P value	Summary
3	8.727	6.658	P<0.001	***
4	7.46	4.276	P<0.001	***
5	4.918	2.818	P < 0.05	*
6	8.591	4.955	P<0.001	***

UT/LV.GFP vs S1.2 contra				
Source of Variation	% of total variation	P value		
Interaction	11.83	0.1632		
hem	5.56	0.1879		
slice	4.87	0.4594		
Source of Variation	P value summary	Significant?		
Interaction	ns	No		
hem	ns	No		
slice	ns	No		
Source of Variation	Df	Sum-of-squares	Mean square	F
Interaction	2	38.06	19.03	1.951
hem	1	17.88	17.88	1.833
slice	2	15.66	7.828	0.8027
Residual	25	243.8	9.753	
Bonferroni posttests				
slice	UT/LV.GFP	S1.2 contra	Difference	95% CI of diff.
3	29.35	34.2	4.851	-0.05594 to 9.758
6	30.19	30.65	0.4575	-4.450 to 5.365
9	32.38	31.95	-0.4267	-6.547 to 5.694
slice	Difference	t	P value	Summary
3	4.851	2.537	P > 0.05	ns
6	0.4575	0.2392	P > 0.05	ns
9	-0.4267	0.1789	P > 0.05	ns

UT/LV.GFP vs S1.3 injected				
Source of Variation	% of total variation	P value		
Interaction	2.16	0.4604		
hem	60.06	< 0.0001		
slice	2.11	0.4699		
Source of Variation	P value summary	Significant?		
Interaction	ns	No		
hem	***	Yes		
slice	ns	No		
Source of Variation	Df	Sum-of-squares	Mean square	F
Interaction	3	43.5	14.5	0.8784
hem	1	1210	1210	73.3
slice	3	42.57	14.19	0.8596
Residual	40	660.3	16.51	
Bonferroni posttests				
slice	UT/LV.GFP	S1.3 injected	Difference	95% CI of diff.
3	29.74	42.72	12.97	7.371 to 18.57
4	29.28	41.05	11.77	4.255 to 19.28
5	29.24	37.87	8.624	1.764 to 15.48
6	30.38	39.22	8.841	3.240 to 14.44
slice	Difference	t	P value	Summary
3	12.97	6.058	P<0.001	***
4	11.77	4.097	P<0.001	***
5	8.624	3.288	P < 0.05	*
6	8.841	4.129	P<0.001	***

UT/LV.GFP vs S1.3 contra				
Source of Variation	% of total variation	P value		
Interaction	6.85	0.1689		
hem	37.45	0.0001		
slice	0.49	0.872		
Source of Variation	P value summary	Significant?		
Interaction	ns	No		
hem	***	Yes		
slice	ns	No		
Source of Variation	Df	Sum-of-squares	Mean square	F
Interaction	2	34.05	17.02	1.911
hem	1	186.2	186.2	20.91
slice	2	2.454	1.227	0.1377
Residual	25	222.7	8.907	
Bonferroni posttests				
UT+P2 NHPs vs S1.3 U17 contra				
slice	UT/LV.GFP	S1.3 contra	Difference	95% CI of diff.
3	29.35	37.4	8.051	3.362 to 12.74
6	30.19	35.53	5.333	0.6429 to 10.02
9	32.38	34.75	2.373	-3.476 to 8.222
slice	Difference	t	P value	Summary
3	8.051	4.405	P<0.001	***
6	5.333	2.918	P < 0.05	*
9	2.373	1.041	P > 0.05	ns

Figure	Statistical analysis										
Fig.5C	**Two-way ANOVA**						**Two-way ANOVA**				
	UT/LV.GFP vs S2.1 injected						UT/LV.GFP vs S2.1 contra				
	Source of Variation	% of total variation	P value				Source of Variation	% of total variation	P value		
	Interaction	0.32	0.9282				Interaction	9.12	0.0613		
	hem	64.46	< 0.0001				hem	43.88	< 0.0001		
	slice	0.36	0.9164				slice	3.58	0.3103		
	Source of Variation	P value summary	Significant?				Source of Variation	P value summary	Significant?		
	Interaction	ns	No				Interaction	ns	No		
	hem	***	Yes				hem	***	Yes		
	slice	ns	No				slice	ns	No		
	Source of Variation	Df	Sum-of-squares	Mean square	F		Source of Variation	Df	Sum-of-squares	Mean square	F
	Interaction	3	15.41	5.137	0.1514		Interaction	2	98.65	49.32	3.128
	hem	1	3099	3099	91.34		hem	1	474.7	474.7	30.1
	slice	3	17.25	5.749	0.1694		slice	2	38.69	19.34	1.227
	Residual	40	1357	33.93			Residual	25	394.2	15.77	
	Bonferroni posttests						***Bonferroni posttests***				
	slice	UT/LV.GFP	S2.1 injected	Difference	95% CI of diff.		slice	UT/LV.GFP	S2.1 contra	Difference	95% CI of diff.
	3	29.74	46.5	16.76	9.352 to 24.16		3	29.35	42.93	13.58	7.337 to 19.82
	4	29.28	48.68	19.39	8.621 to 30.17		6	30.19	36.7	6.508	0.2679 to 12.75
	5	29.24	45	15.76	4.984 to 26.53		9	32.38	37.45	5.073	-2.709 to 12.86
	6	30.38	46.97	16.59	8.561 to 24.62						
	slice	Difference	t	P value	Summary		slice	Difference	t	P value	Summary
	3	16.76	5.92	P<0.001	***		3	13.58	5.583	P<0.001	***
	4	19.39	4.709	P<0.001	***		6	6.508	2.676	P > 0.05	ns
	5	15.76	3.826	P<0.01	**		9	5.073	1.673	P > 0.05	ns
	6	16.59	5.404	P<0.001	***						
	UT/LV.GFP vs S2.2 injected						UT/LV.GFP vs S2.2 contra				
	Source of Variation	% of total variation	P value				Source of Variation	% of total variation	P value		
	Interaction	9.33	0.0217				Interaction	6.48	0.1947		
	TREATMENT	28.46	< 0.0001				hem	22.53	< 0.0001		
	SLICES	10.18	< 0.0001				slice	10.7	0.0276		
	Source of Variation	P value summary	Significant?				Source of Variation	P value summary	Significant?		
	Interaction	*	Yes				Interaction	ns	No		
	TREATMENT	***	Yes				hem	***	Yes		
	SLICES	***	Yes				slice	*	Yes		
	Source of Variation	Df	Sum-of-squares	Mean square	F		Source of Variation	Df	Sum-of-squares	Mean square	F
	Interaction	28	1592	56.85	1.678		Interaction	7	277.8	39.69	1.458
	TREATMENT	4	4859	1215	35.85		hem	1	966.4	966.4	35.5
	SLICES	7	1738	248.2	7.327		slice	7	459	65.57	2.409
	Residual	225	7622	33.88			Residual	78	2123	27.22	
	Bonferroni posttests						***Bonferroni posttests***				
	SLICES	UT+P2 NHPs	S2.2 INJ hemisph	Difference	95% CI of diff.		slice	UT+P2 NHPs	S2.2 CONTRAL her	Difference	95% CI of diff.
	2	29.92	39.19	9.265	-5.952 to 24.48		2	29.92	33.59	3.668	-8.304 to 15.64
	3	29.74	50.9	21.15	12.10 to 30.21		3	29.74	38.7	8.954	1.226 to 16.68
	4	29.28	43.9	14.62	3.419 to 25.82		4	29.28	35.19	5.908	-3.557 to 15.37
	5	29.24	44.11	14.87	3.189 to 26.55		5	29.24	35.51	6.267	-3.198 to 15.73
	6	30.38	38.29	7.914	-0.4621 to 16.29		6	30.38	36.99	6.616	-1.112 to 14.34
	7	31.15	38.33	7.18	-1.038 to 15.40		7	31.15	33.43	2.284	-5.048 to 9.616
	8	31.71	45.91	14.2	1.342 to 27.06		8	31.71	38.73	7.02	-3.349 to 17.39
	9	32.38	42	9.619	-3.991 to 23.23		9	32.38	48.18	15.8	5.433 to 26.17
	SLICES	Difference	t	P value	Summary		slice	Difference	t	P value	Summary
	2	9.265	1.95	P > 0.05	ns		2	3.668	0.8611	P > 0.05	ns
	3	21.15	7.48	P<0.001	***		3	8.954	3.256	P < 0.05	*
	4	14.62	4.179	P<0.001	***		4	5.908	1.754	P > 0.05	ns
	5	14.87	4.076	P<0.001	***		5	6.267	1.861	P > 0.05	ns
	6	7.914	3.025	P < 0.05	*		6	6.616	2.406	P > 0.05	ns
	7	7.18	2.797	P < 0.05	*		7	2.284	0.8756	P > 0.05	ns
	8	14.2	3.536	P<0.01	**		8	7.02	1.903	P > 0.05	ns
	9	9.619	2.263	P > 0.05	ns		9	15.8	4.283	P<0.001	***
	UT/LV.GFP vs S2.3 injected						UT/LV.GFP vs S2.3 contra				
	Source of Variation	% of total variation	P value				Source of Variation	% of total variation	P value		
	Interaction	1.18	0.6394				Interaction	0.03	0.9893		
	hem	66.45	< 0.0001				hem	46.7	< 0.0001		
	slice	0.66	0.8114				slice	4.23	0.2795		
	Source of Variation	P value summary	Significant?				Source of Variation	P value summary	Significant?		
	Interaction	ns	No				Interaction	ns	No		
	hem	***	Yes				hem	***	Yes		
	slice	ns	No				slice	ns	No		
	Source of Variation	Df	Sum-of-squares	Mean square	F		Source of Variation	Df	Sum-of-squares	Mean square	F
	Interaction	3	34.05	11.35	0.5678		Interaction	2	0.3752	0.1876	0.01072
	hem	1	1913	1913	95.71		hem	1	519	519	29.67
	slice	3	19.14	6.38	0.3192		slice	2	46.97	23.48	1.342
	Residual	41	819.5	19.99			Residual	25	437.4	17.5	
	Bonferroni posttests						***Bonferroni posttests***				
	slice	UT/LV.GFP	S2.3 injected	Difference	95% CI of diff.		slice	UT/LV.GFP	S2.3 contra	Difference	95% CI of diff.
	3	29.74	41.55	11.81	5.649 to 17.96		3	29.35	37.9	8.551	1.979 to 15.12
	4	29.28	45.68	16.4	8.135 to 24.65		6	30.19	38.83	8.633	2.060 to 15.21
	5	29.24	42.34	13.1	5.942 to 20.25		9	32.38	41.5	9.123	0.9258 to 17.32
	slice	Difference	t	P value	Summary		slice	Difference	t	P value	Summary
	3	11.81	5.01	P<0.001	***		3	8.551	3.338	P < 0.05	*
	4	16.4	5.186	P<0.001	***		6	8.633	3.37	P<0.01	**
	5	13.1	4.783	P<0.001	***		9	9.123	2.856	P < 0.05	*

Figure	Statistical analysis					
**Fig.5D**	**One-way analysis of variance**					
	P value	< 0.0001				
	P value summary	***				
	Are means signif. different? (P < 0.05)	Yes				
	Number of groups	5				
	F	50.61				
	R squared	0.3488				
	ANOVA Table	SS	df	MS		
	Treatment (between columns)	8173	4	2043		
	Residual (within columns)	15260	378	40.37		
	Total	23430	382			
	Tukey's Multiple Comparison Test	Mean Diff.	q	Significant? P < 0.05?	Summary	95% CI of diff
	normal controls vs Study group 1 inj	-9.979	13.01	Yes	***	-12.98 to -6.979
	normal controls vs Study group 1 contra	-2.141	2.418	No	ns	-5.605 to 1.323
	normal controls vs Stoudy group 2 inj	-13.12	17.05	Yes	***	-16.13 to -10.11
	normal controls vs Study group 2 contra	-7.533	9.161	Yes	***	-10.75 to -4.317
	Study group 1 inj vs Study group 1 contra	7.838	10.29	Yes	***	4.857 to 10.82
	Study group 1 inj vs Stoudy group 2 inj	-3.139	5.038	Yes	**	-5.576 to -0.7017
	Study group 1 inj vs Study group 2 contra	2.445	3.557	No	ns	-0.2440 to 5.134
	Study group 1 contra vs Stoudy group 2 inj	-10.98	14.36	Yes	***	-13.97 to -7.987
	Study group 1 contra vs Study group 2 contra	-5.392	6.595	Yes	***	-8.591 to -2.194
	Stoudy group 2 inj vs Study group 2 contra	5.584	8.091	Yes	***	2.885 to 8.284

**Fig.5E**	**One-way analysis of variance**					
	P value	0.0422				
	P value summary	*				
	Are means signif. different? (P < 0.05)	Yes				
	Number of groups	3				
	F	3.461				
	R squared	0.1613				
	ANOVA Table	SS	df	MS		
	Treatment (between columns)	379.7	2	189.8		
	Residual (within columns)	1975	36	54.85		
	Total	2354	38			
	Dunnett's Multiple Comparison Test	Mean Diff.	q	Significant? P < 0.05?	Summary	95% CI of diff
	Pilot group vs Study group 1	-7.57	2.592	Yes	*	-14.30 to -0.8406
	Pilot group vs Study group 2	-3.896	1.375	No	ns	-10.43 to 2.634

Figure	Statistical analysis				
**Fig.7A**	**Two-way ANOVA**				
	Source of Variation	% of total variation	P value		
	Interaction	23.05	0.0057		
	treat	22.33	< 0.0001		
	slice	21.24	< 0.0001		
	Source of Variation	P value summary	Significant?		
	Interaction	**	Yes		
	treat	***	Yes		
	slice	***	Yes		
	Source of Variation	Df	Sum-of-squares	Mean square	F
	Interaction	16	10.15	0.6341	2.608
	treat	4	9.827	2.457	10.1
	slice	4	9.351	2.338	9.615
	Residual	46	11.18	0.2431	
	Bonferroni posttests				
	UT WT vs JT02 inj				
	slice	UT WT	JT02 inj	Difference	95% CI of diff.
	5	1.77	1.235	-0.535	-2.112 to 1.042
	7	2.32	1.28	-1.04	-2.972 to 0.8917
	9	2.033	0.79	-1.243	-2.447 to -0.03785
	11	1.933	1.757	-0.1758	-1.380 to 1.029
	15	1.603	1.42	-0.1825	-1.548 to 1.183
	slice	Difference	t	P value	Summary
	5	-0.535	1.085	P > 0.05	ns
	7	-1.04	1.722	P > 0.05	ns
	9	-1.243	3.299	P<0.01	**
	11	-0.1758	0.4669	P > 0.05	ns
	15	-0.1825	0.4274	P > 0.05	ns
	UT WT vs JT02 contra				
	slice	UT WT	JT02 contra	Difference	95% CI of diff.
	5	1.77	0.855	-0.915	-2.492 to 0.6623
	7	2.32	0.71	-1.61	-3.187 to -0.03274
	9	2.033	1.144	-0.8885	-1.947 to 0.1696
	11	1.933	1.79	-0.1425	-1.258 to 0.9728
	15	1.603	1.685	0.0825	-1.283 to 1.448
	slice	Difference	t	P value	Summary
	5	-0.915	1.856	P > 0.05	ns
	7	-1.61	3.265	P < 0.05	*
	9	-0.8885	2.686	P > 0.05	ns
	11	-0.1425	0.4087	P > 0.05	ns
	15	0.0825	0.1932	P > 0.05	ns
	UT WT vs JV02 inj				
	slice	UT WT	JV02 inj	Difference	95% CI of diff.
	5	1.77	2.11	0.34	-1.237 to 1.917
	7	2.32	1.03	-1.29	-3.222 to 0.6417
	9	2.033	1.56	-0.4725	-1.531 to 0.5856
	11	1.933	2.273	0.3408	-0.8638 to 1.545
	15	1.603	2.725	1.123	-0.2434 to 2.488
	slice	Difference	t	P value	Summary
	5	0.34	0.6895	P > 0.05	ns
	7	-1.29	2.136	P > 0.05	ns
	9	-0.4725	1.428	P > 0.05	ns
	11	0.3408	0.905	P > 0.05	ns
	15	1.123	2.629	P > 0.05	ns
	UT WT vs JV02 contra				
	slice	UT WT	JV02 contra	Difference	95% CI of diff.
	5	1.77	1.78	0.01	-1.430 to 1.450
	7	2.32	1.41	-0.91	-2.842 to 1.022
	9	2.033	1.868	-0.1642	-1.182 to 0.8539
	11	1.933	3.775	1.843	0.7272 to 2.958
	15	1.603	2.8	1.198	-0.1684 to 2.563
	slice	Difference	t	P value	Summary
	5	0.01	0.02222	P > 0.05	ns
	7	-0.91	1.507	P > 0.05	ns
	9	-0.1642	0.5158	P > 0.05	ns
	11	1.843	5.284	P<0.001	***
	15	1.198	2.804	P < 0.05	*

Fig.7B	One-way analysis of variance				
	P value	< 0.0001			
	P value summary	***			
	Are means signif. different? (P < 0.05)	Yes			
	Number of groups	3			
	F	18.26			
	R squared	0.3824			
	Bartlett's test for equal variances				
	Bartlett's statistic (corrected)	23.54			
	P value	< 0.0001			
	P value summary	***			
	Do the variances differ signif. (P < 0.05	Yes			
	ANOVA Table	SS	df	MS	95% CI of diff
	Treatment (between columns)	23.25	2	11.63	-1.452 to -0.2465
	Residual (within columns)	37.56	59	0.6366	-0.02271 to 1.162
	Total	60.81	61		
	Bonferroni's Multiple Comparison Te	Mean Diff.	t	Significant? P	Summary
	wt vs JV02	-0.8494	3.24	Yes	**
	wt vs JT02	0.5696	2.212	No	ns

Fig.7C	One-way analysis of variance				
	P value	0.0227			
	P value summary	*			
	Are means signif. different? (P < 0.05)	Yes			
	Number of groups	3			
	F	7.598			
	R squared	0.7169			
	ANOVA Table	SS	df	MS	95% CI of diff
	Treatment (between columns)	1.882	2	0.941	-0.3726 to 1.273
	Residual (within columns)	0.7431	6	0.1239	0.2907 to 1.936
	Total	2.625	8		
	Dunnett's Multiple Comparison Test	Mean Diff.	q	Significant? P	Summary
	SC WT vs SC JV02	0.45	1.566	No	ns
	SC WT vs SC JT02	1.113	3.874	Yes	*

Fig.7F	Two-way ANOVA				
	Source of Variation	% of total variation	P value		
	Interaction	1.12	0.7746		
	genotype	41.05	< 0.0001		
	age	1.83	0.5748		
	Source of Variation	P value summary	Significant?		
	Interaction	ns	No		
	genotype	***	Yes		
	age	ns	No		
	Source of Variation	Df	Sum-of-squares	Mean square	F
	Interaction	4	5.372	1.343	0.4466
	genotype	1	196.2	196.2	65.25
	age	4	8.764	2.191	0.7285
	Residual	89	267.6	3.007	
	Bonferroni posttests				
	Normal vs Affected				
	age	Normal	Affected	Difference	95% CI of diff.
	2m	6.9	3.75	-3.15	-4.817 to -1.483
	3m	7.8	4.18	-3.62	-5.388 to -1.852
	4m	8.23	4.54	-3.69	-5.943 to -1.437
	5m	8.5	3.9	-4.6	-7.364 to -1.836
	6m	9	4.07	-4.93	-9.810 to -0.05013
	age	Difference	t	P value	Summary
	2m	-3.15	4.975	P<0.001	***
	3m	-3.62	5.39	P<0.001	***
	4m	-3.69	4.311	P<0.001	***
	5m	-4.6	4.381	P<0.001	***
	6m	-4.93	2.659	P < 0.05	*

Figure	Statistical analysis					
Fig.EV2 C	**One-way analysis of variance**					
	P value	< 0.0001				
	P value summary	***				
	Are means signif. different? (P < 0.05)	Yes				
	Number of groups	5				
	F	324.2				
	R squared	0.9767				
	Bartlett's test for equal variances					
	Bartlett's statistic (corrected)	2.273				
	P value	0.6857				
	P value summary	ns				
	Do the variances differ signif. (P < 0.05)	No				
	ANOVA Table	SS	df	MS		
	Treatment (between columns)	1655	4	413.7		
	Residual (within columns)	39.55	31	1.276		
	Total	1694	35			
	Bonferroni's Multiple Comparison Test	Mean Diff.	t	Significant? P	Summary	95% CI of diff
	P2 vs S1.1 injected	-17.72	31.38	Yes	***	-19.43 to -16.01
	P2 vs S1.1 contralateral	-12.71	22.51	Yes	***	-14.42 to -11.01
	P2 vs S2.2 injected	-12.32	21.81	Yes	***	-14.03 to -10.61
	P2 vs S2.2 contra	-12.64	22.38	Yes	***	-14.34 to -10.93
	S1.1 injected vs S1.1 contralateral	5.008	7.68	Yes	***	3.038 to 6.979
	S1.1 injected vs S2.2 injected	5.402	8.283	Yes	***	3.431 to 7.372
	S1.1 injected vs S2.2 contra	5.085	7.797	Yes	***	3.114 to 7.056
	S1.1 contralateral vs S2.2 injected	0.3933	0.6031	No	ns	-1.577 to 2.364
	S1.1 contralateral vs S2.2 contra	0.07667	0.1176	No	ns	-1.894 to 2.047
	S2.2 injected vs S2.2 contra	-0.3167	0.4856	No	ns	-2.288 to 1.654

Figure	Statistical analysis					
Fig.EV5						
	**One-way analysis of variance**					
	P value	0.0225				
	P value summary	*				
	Are means signif. different? (P < 0.05)	Yes				
	Number of groups	7				
	F	3.258				
	R squared	0.5071				
	ANOVA Table	SS	df	MS		
	Treatment (between columns)	711.4	6	118.6		
	Residual (within columns)	691.4	19	36.39		
	Total	1403	25			
	Dunnett's Multiple Comparison Test	Mean Diff.	q	Significant? P	Summary	95% CI of diff
	KD UT vs JT02 slice 5 inj.	-2.84	0.5157	No	ns	-18.34 to 12.66
	KD UT vs JT02 slice 7 inj.	-2.343	0.5494	No	ns	-14.35 to 9.664
	KD UT vs JT02 slice 9 inj.	-1.463	0.3174	No	ns	-14.43 to 11.51
	KD UT vs JT02 slice 11 inj.	-17.59	3.571	Yes	*	-31.45 to -3.722
	KD UT vs JT02 slice 13 inj.	-2.688	0.5833	No	ns	-15.66 to 10.28
	KD UT vs JT02 slice 15 inj.	0.085	0.01845	No	ns	-12.88 to 13.05

Figure	Statistical analysis			
Table 2	**Kruskal-Wallis test**			
	P value	0.0761		
	Exact or approximate P value?	Gaussian Approximation		
	P value summary	ns		
	Do the medians vary signif. (P < 0.05)	No		
	Number of groups	3		
	Kruskal-Wallis statistic	5.152		
	Dunn's Multiple Comparison Test	Difference in rank sum	Significant? P < 0.05?	Summary
	Pilot group inj vs Study group 2 inj	-1.917	No	ns
	Study group 1 inj vs Study group 2 inj	-4.5	Yes	*
	**Kruskal-Wallis test**			
	P value	0.0513		
	Exact or approximate P value?	Gaussian Approximation		
	P value summary	ns		
	Do the medians vary signif. (P < 0.05)	No		
	Number of groups	3		
	Kruskal-Wallis statistic	5.939		
	Dunn's Multiple Comparison Test	Difference in rank sum	Significant? P < 0.05?	Summary
	Pilot group tot vs Study group 2 tot	-2.083	No	ns
	Study group 1 tot vs Study group 2 tot	-4.833	Yes	*

References

Annoni A, Brown BD, Cantore A, Sergi LS, Naldini L, Roncarolo MG (2009) In vivo delivery of a microRNA-regulated transgene induces antigen-specific regulatory T cells and promotes immunologic tolerance. Blood 114**:** 5152-5161

Bayley A (2000) Narrowing the gap. J Med Ethics 26**:** 51-53

Brazelton TB (1973) Assessment of the infant at risk. Clin Obstet Gynecol 16**:** 361-375

Champoux M, Bennett A, Shannon C, Higley JD, Lesch KP, Suomi SJ (2002a) Serotonin transporter gene polymorphism, differential early rearing, and behavior in rhesus monkey neonates. Mol Psychiatry 7**:** 1058-1063

Champoux M, Hibbeln JR, Shannon C, Majchrzak S, Suomi SJ, Salem N, Jr., Higley JD (2002b) Fatty acid formula supplementation and neuromotor development in rhesus monkey neonates. Pediatric research 51**:** 273-281

Champoux M, Higley JD, Suomi SJ (1997) Behavioral and physiological characteristics of Indian and Chinese-Indian hybrid rhesus macaque infants. Dev Psychobiol 31**:** 49-63

Champoux M, Suomi SJ, Schneider ML (1994) Temperament differences between captive Indian and Chinese-Indian hybrid rhesus macaque neonates. Lab Anim Sci 44**:** 351-357

Lattanzi A, Neri M, Maderna C, di Girolamo I, Martino S, Orlacchio A, Amendola M, Naldini L, Gritti A (2010) Widespread enzymatic correction of CNS tissues by a single intracerebral injection of therapeutic lentiviral vector in leukodystrophy mouse models. Human molecular genetics 19**:** 2208-2227

Neri M, Ricca A, di Girolamo I, Alcala'-Franco B, Cavazzin C, Orlacchio A, Martino S, Naldini L, Gritti A (2011) Neural stem cell gene therapy ameliorates pathology and function in a mouse model of globoid cell leukodystrophy. Stem Cells 29**:** 1559-1571
