# Supplementary material for: Pervasive supply of therapeutic lysosomal enzymes in the CNS of normal and Krabbe‐affected non‐human primates by intracerebral lentiviral gene therapy
Source: EMBO Mol Med. 2016 Mar 29;8(5):489–510. doi: 10.15252/emmm.201505850 (PMC5128736; doi:10.15252/emmm.201505850)
Supplement: Supplementary file 2 — Expanded View Figures PDF [file EMMM-8-489-s002.pdf]

Expanded View Figures

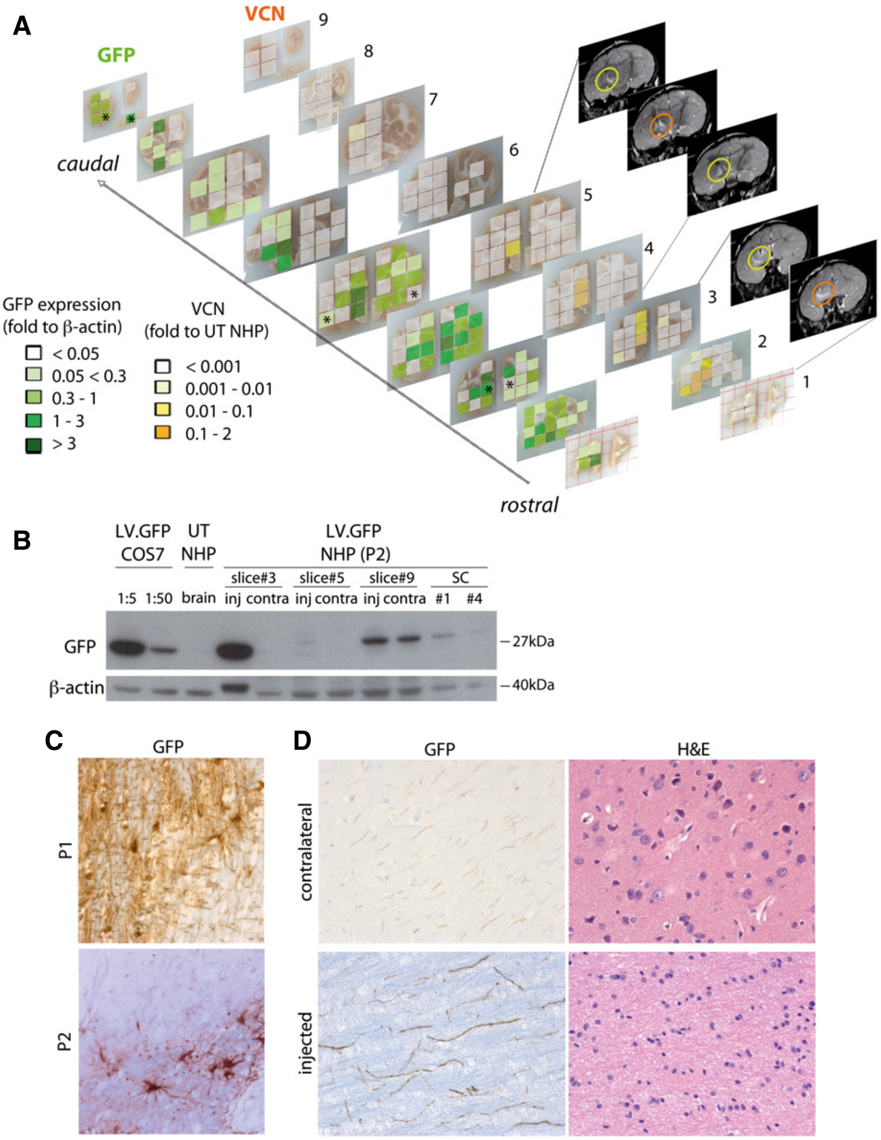

Figure EV1. VCN and GFP distribution in LV.GFP-injected NHPs.

A Vector copy number (VCN) cartography shows integrated LV genome along the rostrocaudal axis (slices 1–9) in LV.GFP-injected P2 NHP assessed by qPCR. Grading of colors ranged from white (VCN < 0.001; corresponding to CT > 37) to dark orange (VCN = 0.1–2). The highest VCN is found in close correspondence to the injection sites, as confirmed by comparison with post-surgery MR images (yellow and orange circles indicate viral suspension close to the injection sites). Cartography shows GFP expression in different blocks of all brain slices based on Western blot analysis. Values are expressed as fold to β-actin and are given a color code according to increasing GFP expression. Asterisks in slices 3, 5, and 9 indicate blocks for which GFP expression is shown in (B).

B Representative Western blot showing GFP expression in selected tissue blocks of the injected and contralateral hemispheres from brain slices 3, 5, and 9 and of SC (blocks #1 and #4). β-actin was used as a housekeeping gene. Positive controls (lanes 1 and 2): LV.GFP-transduced COS7 cells (VCN = 1; 1:5 and 1:50 dilution). Negative control: brain tissue from untreated U14 NHP.

C Representative bright field pictures showing transduced cells at the injection sites of P1 and P2 NHP expressing GFP in cell bodies and processes, as assessed by IHC.

D Representative pictures showing GFP-positive neuronal cell processes in the injected and contralateral hemisphere of LV.GFP-injected NHP. Note the normal tissue architecture assessed on adjacent sections by hematoxylin/eosin staining (H&E).

Figure EV2. ARSA enzymatic activity in CNS tissues of LV.hARSA-injected NHP.

A Percentage of blocks in LV.ARSA-injected NHP in study group 1 (n = 3) and study group 2 (n = 3) grouped according to the extent of supraphysiological ARSA activity. Data are expressed as mean ± SEM.

B Chromatographic diagrams showing overexpression of ARSA activity in injected and contralateral brain hemispheres in representative brain slices of LV.hARSA-injected S1.1 and S2.2 NHP when compared to LV.GFP-treated counterparts (P2). Similar amount of sample proteins were separated by ionic exchange chromatography on DEAE-cellulose and assayed for the ARSA activity using the artificial fluorogenic substrate MUS in the presence (-●-) or absence (-○-) of 125 μM $AgNO_3$ (specific ARSA inhibitor). One unit is defined as the amount of enzyme required to convert 1 nmol of substrate hydrolyzed in 1 min at 37°C.

C ARSA activity measured toward the sulfatide (Morena *et al*, 2014) in representative slices of LV.hARSA-injected NHP S1.1 and S2.2 and LV.GFP-injected NHP (P2). Data are expressed as the mean ± SEM; n = 12 blocks (P2) and 6 blocks/group (S1.1 and S2.2). One-way ANOVA and Bonferroni's multiple comparison test. $^{\S}P < 0.001$ versus P2; $^{\#}P < 0.01$ versus S1.1 injected.

© 2016 The Authors

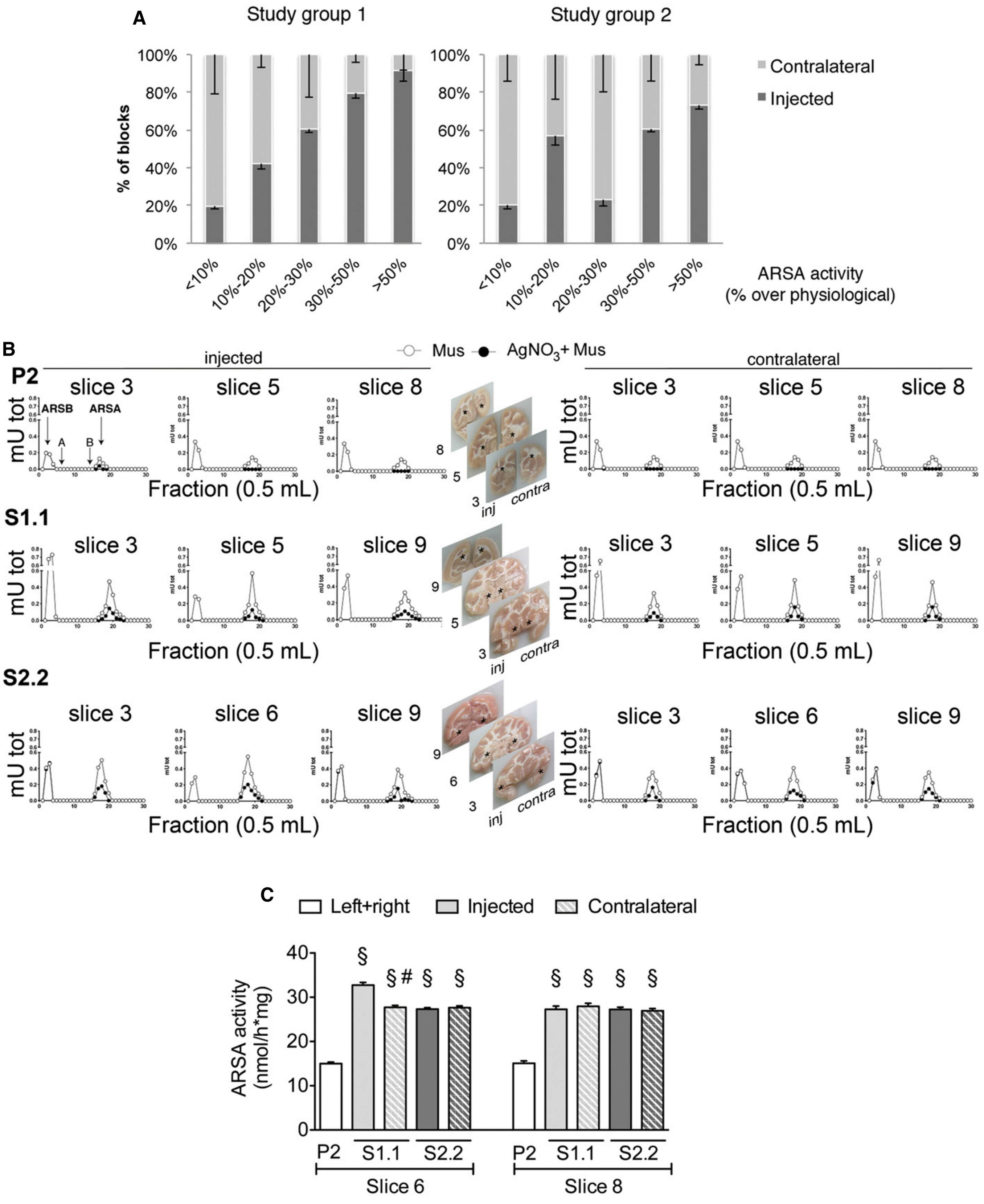

Figure EV2.

 © 2016 The Authors

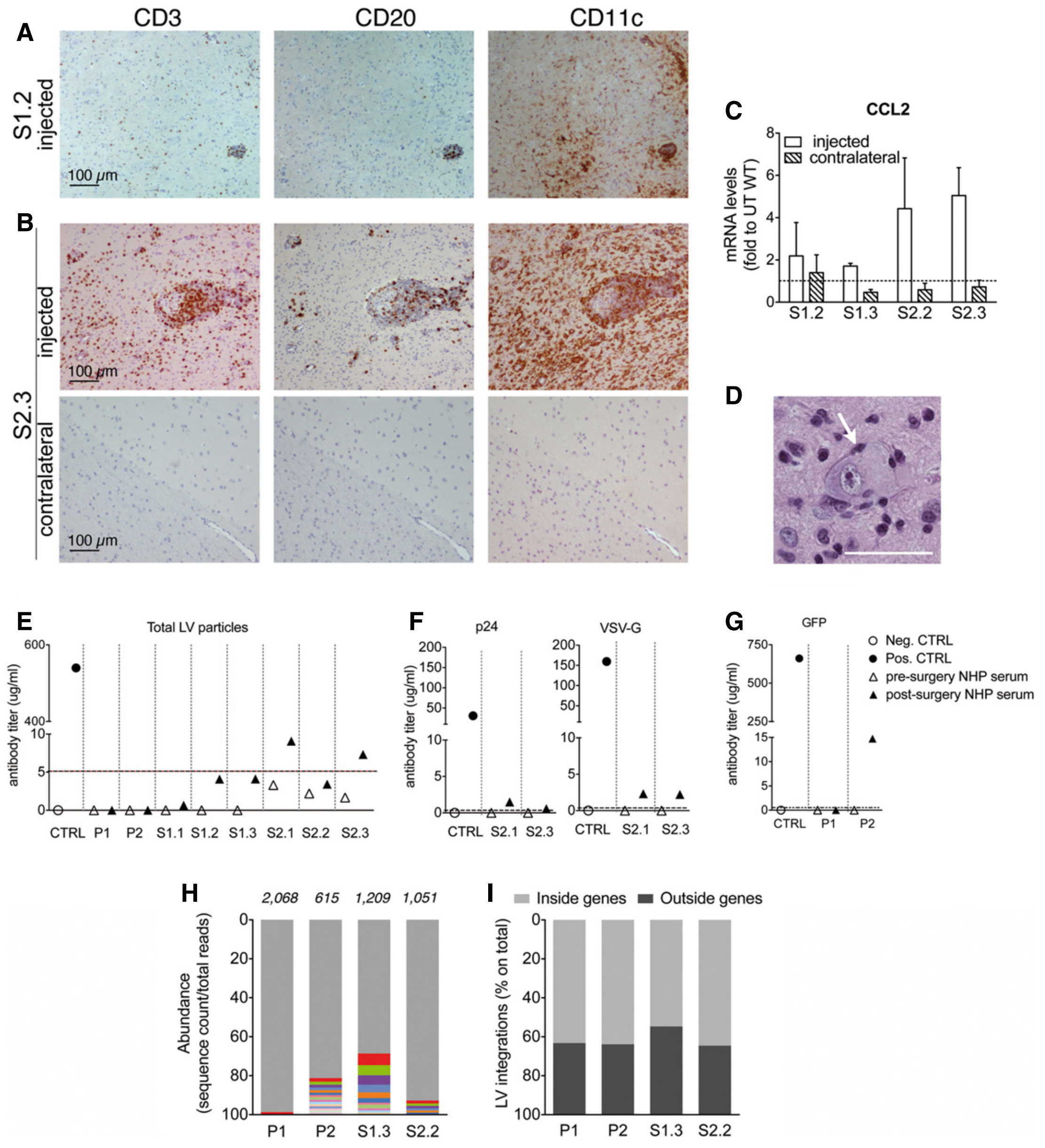

Figure EV3. Favorable safety profile of LV-mediated intracerebral gene therapy.

A, B Local infiltration of $CD3^+$ T cells, $CD20^+$ B cells, and $CD11c^+$ macrophages at the injection site of S1.2 NHP (A) and S2.3 NHP (B). Staining performed on the matched block of the contralateral hemisphere of S2.3 NHP has normal appearance and no detectable signs of inflammation (B). Scale bar, 100 μm.

C CCL2 mRNA expression levels in the injected and contralateral hemispheres of LV.hARSA-injected NHP of study group 1 (S1.2 and S1.3) and study group 2 (2.2 and 2.3). Values are expressed as fold to physiological (dotted line = 1), assessed in matched samples from untreated (UT) WT NHP. Data are expressed as mean ± SEM; n = 2 blocks/hemisphere/animal.

D Neuron with large cellular body, homogenous eosinophilic cytoplasm (interpreted as intracytoplasmic accumulations) and lateral displacement of nucleus (arrows). Scale bar, 40 μm.

E Antibodies against total LV particles in the pre- and post-surgery sera (1:200 dilution) of LV-injected NHPs. Values plotted in the graph are the mean of n = 5 technical replicates. Threshold (dotted line; 5.12 μg/ml) is set as the average titer measured in pre-surgery sera plus three standard deviations.

F Anti-p24 and anti-VSV-G antibodies in the pre- and post-surgery sera (1:20 dilution) of S2.1 and S2.3 NHP. Values plotted in the graph are the mean of n = 3 technical replicates. Threshold: 0 μg/ml.

G Anti-GFP antibodies in the pre- and post-surgery sera (1:200 dilution) of pilot group NHP. Threshold: 0 μg/ml. Antibody titers are expressed in μg/ml. Sera of adult BALB/c mice immunized by systemic injection of LV.ET.GFP.miR-142 (Annoni *et al*, 2009) were used as positive controls. Sera of untreated mice were used as negative controls.

H, I Polyclonal LV integration profile in CNS tissues of LV-injected NHPs. Clonal abundance is calculated as the sequencing reads representing each integration site within each data set. Gray bars represent all vector integrations with an abundance of < 1% (H). Relative proportions of LV integrations mapping inside and outside genes are indicated (I).

© 2016 The Authors

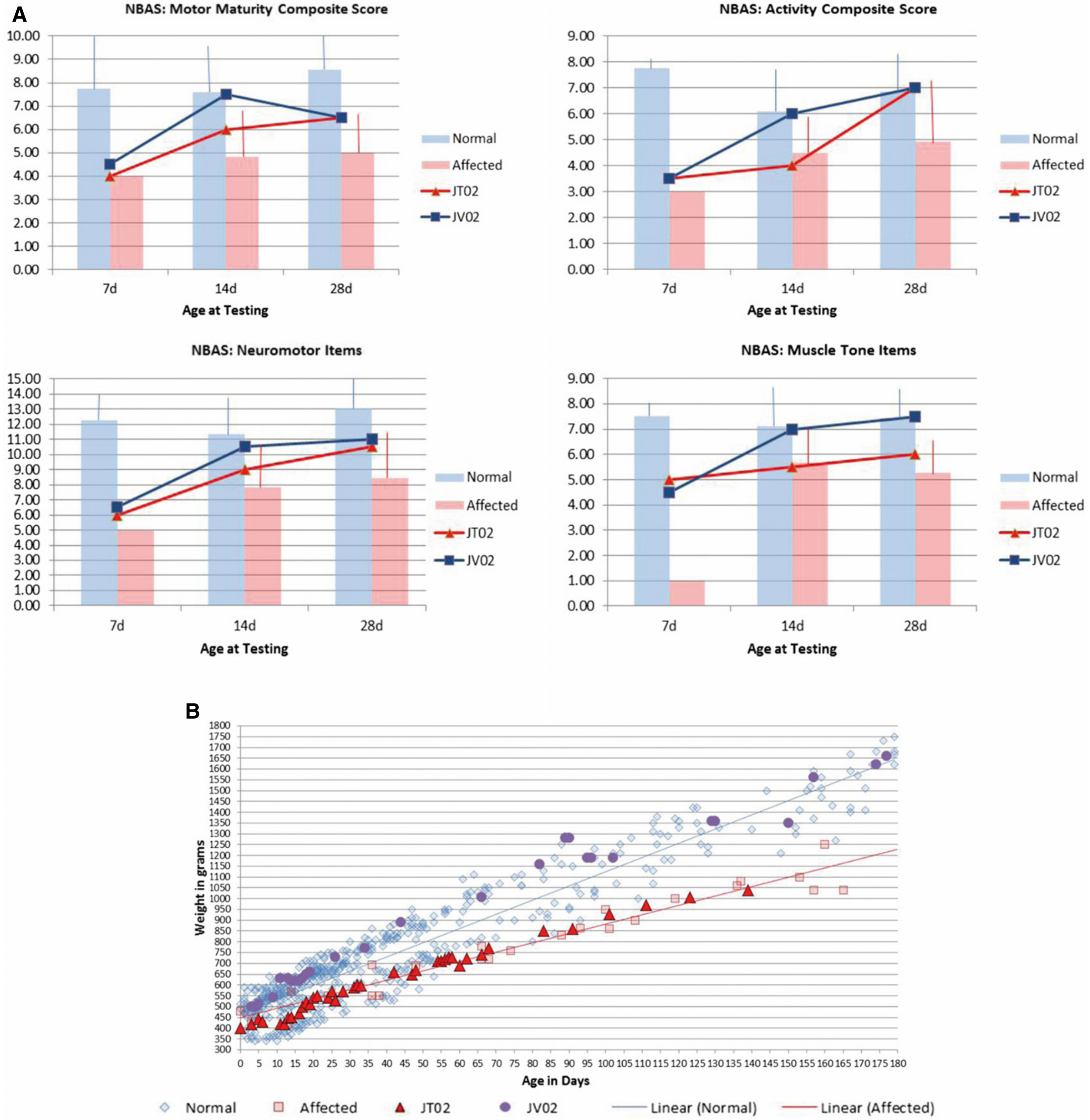

Figure EV4. Pre-treatment behavioral assessment and post-treatment follow-up of LV.hGALC-injected NHP.

A Prior to treatment (at 7, 14 and 28 postnatal days), the study animals JT02 (Krabbe affected) and JV02 (normal) underwent a neurobehavioral assessment using a non-human primate adaptation of the Neurobehavioral Assessment Scale (NBAS). Composite scores for the 2 study animals as well as historical data collected on genetically normal animals and untreated Krabbe-affected juveniles are presented. With the exception of the 7-day testing period, scores for study animals fell within historical ranges for genetic status and age. In particular, JT02 scored lower on motor maturity, activity, neuromotor items, and muscle tone items at each age as compared to unaffected animals. Historical data are presented as means plus one standard deviation. Affected animals: $n = 1$, $n = 6$, $n = 7$ at 7d, 14d and 28d, respectively. Normal animals: $n = 2$, $n = 16$, $n = 22$ at 7d, 14d and 28d, respectively. See also Appendix Table S8.

B Body weight of JT02 and JV02 are comparable to weight from animals of similar genetic status and age.

 © 2016 The Authors

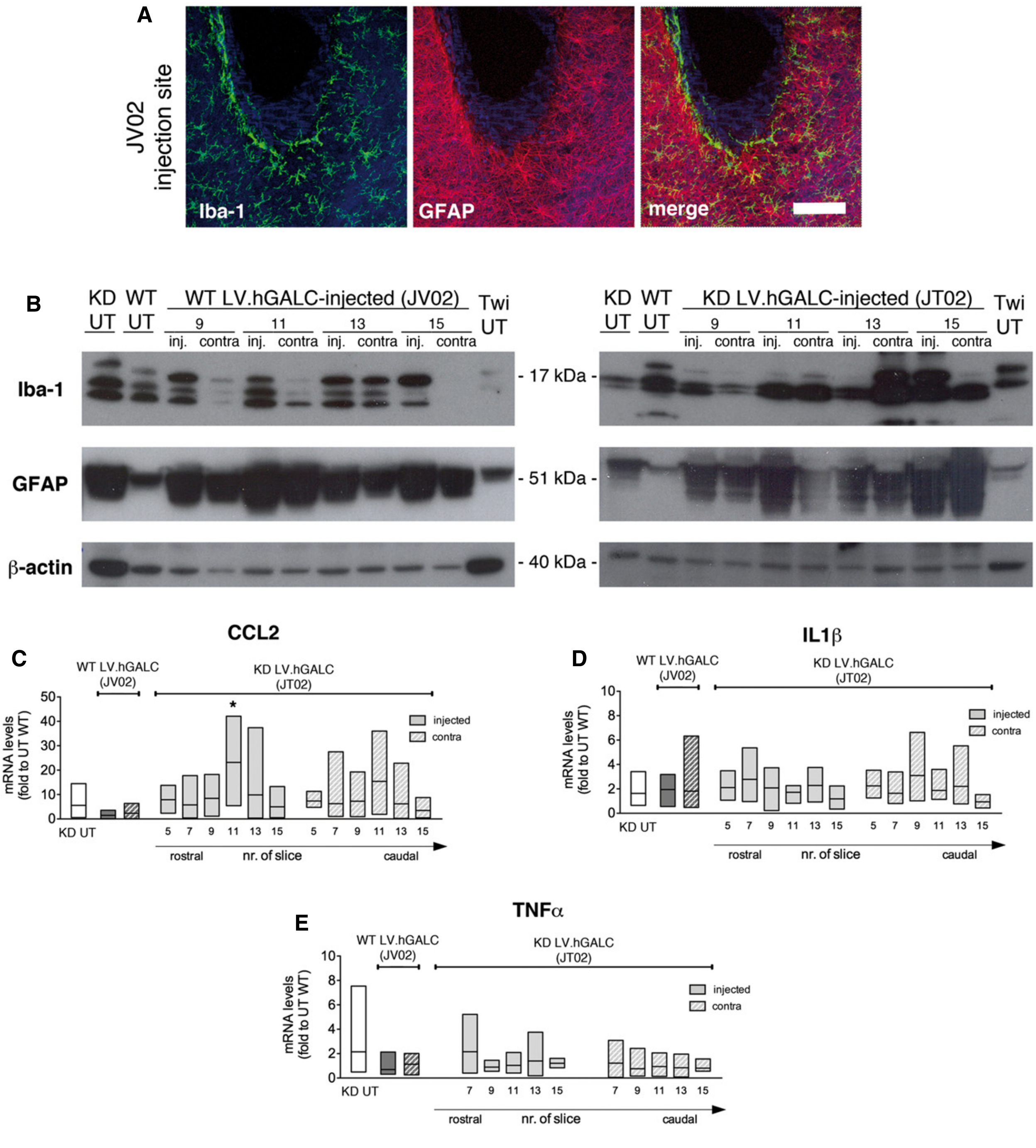

Figure EV5. Local brain inflammation in LV.hGALC-injected NHP.

A Representative confocal images showing microgliosis (Iba-1, green) and astrogliosis (GFAP, red) in the vicinity of the injection sites (slice 10) in LV.hGALC-injected NHP (JV02). Nuclei counterstained with ToPro (blue). Scale bar: 80 µm.

B Western blots showing GFAP and Iba-1 expression in selected tissue blocks from brain slices 9, 11, 13, and 15 (injected and contralateral hemispheres) of JV02 and LV.hGALC-injected Krabbe-affected NHP (JT02). β-actin was used as a housekeeping gene. Brain tissues from untreated (UT) WT and Krabbe NHP, and Twitcher (Twi) mouse brain were used as controls.

C–E mRNA expression levels of CCL2 (C), interleukin-1β (D) and TNF-α (E) along the rostrocaudal axis of JV02 and JT02 (brain slices 5–17; injected and contralateral hemispheres; n = 6–12 blocks/animal). Brain samples from UT WT and Krabbe-affected NHP were used as controls. Data in (C, D) are plotted as floating bars (line at mean) and analyzed by one-way ANOVA and Dunnet's multiple comparison test, $^*P < 0.05$ versus KD UT.

© 2016 The Authors
